# Fabrication and temperature-dependent electrical characterization of a C-shape nanowire patterned by a DNA origami

**DOI:** 10.1038/s41598-021-81178-8

**Published:** 2021-01-21

**Authors:** Türkan Bayrak, Amanda Martinez-Reyes, David Daniel Ruiz Arce, Jeffrey Kelling, Enrique C Samano, Artur Erbe

**Affiliations:** 1 Institute of Ion Beam Physics and Materials Research, Helmholtz-Zentrum Dresden-Rossendorf, 01328 Dresden, Germany; 2 Cluster of Excellence Center for Advancing Electronics Dresden (cfaed), TU Dresden, 01062 Dresden, Germany; 3 Centro de Nanociencias y Nanotecnologia, Universidad Nacional Autonoma de Mexico, Ensenada, BC Mexico; 4 Department of Information Services and Computing, Helmholtz-Zentrum Dresden-Rossendorf, 01314 Dresden, Germany; 5 Present Address: Energy Transition Lab., Faculty of Technology, Policy and Management, TU Delft, Delft, Netherlands

**Keywords:** Electronic devices, Nanowires

## Abstract

We introduce a method based on directed molecular self-assembly to manufacture and electrically characterise C-shape gold nanowires which clearly deviate from typical linear shape due to the design of the template guiding the assembly. To this end, gold nanoparticles are arranged in the desired shape on a DNA-origami template and enhanced to form a continuous wire through electroless deposition. C-shape nanowires with a size below 150nm on a ${\hbox {SiO}_2}/\hbox {Si}$ substrate are contacted with gold electrodes by means of electron beam lithography. Charge transport measurements of the nanowires show hopping, thermionic and tunneling transports at different temperatures in the 4.2K to 293K range. The different transport mechanisms indicate that the C-shape nanowires consist of metallic segments which are weakly coupled along the wires.

## Introduction

DNA templates are powerful tools for guiding the assembly of nanostructures as building blocks into defined patterns with a high degree of complexity^1–5^. Many kinds of devices or functional systems may take advantage of this approach including sensors, nanoelectronic devices and surface plasmon-based circuits^6–10^. DNA origami in particular is a versatile construction material within the self-assembly of complex systems, allowing, e.g., the generation of nanoscale electronic components for future applications. DNA double strands^11,12^ and DNA origami guided self-assembly provides the possibility to precisely control the assembly of metallic^13^, semiconducting^14–16^ or magnetic^17^ nanoparticles (NPs) into elongated wires via Watson–Crick base pairing^18^. Nanoparticles can be attached at specific sites by functionalizing DNA single strands at the desired location of the origami structures either chemically or by extending staple strands with additional nucleotides that serve as sticky ends for complementary sequences. Fully metallic gold wires can be produced this way by metallizing an array of gold NPs^19–23^.

The degree of miniaturization achievable through DNA origami guided assembly of gold nanowires, however, is still to be determined. Here we explore the feasibility of the DNA origami technique for creating nanodevices within the current size limits by fabricating a split-ring resonator (SRR), a tiny LC circuit^24^. The main application of this design is the generation of a metamaterial^25^. The inductor in a SRR is brought about by an incident electromagnetic wave which produces a time dependent electromotive force. The topology of the SRR corresponds to two C-shape concentric wires with a slit (capacitor) on opposite sides having radii $r_{\mathrm{ext}}$ and $r_{\mathrm{int}}$, with $r_{\mathrm{ext}} > r_{\mathrm{int}}$, and a gap *d* between the perimeter of the wires^26^. As a proof of concept of this device, a C-shape Au nanowire is used as a case study to achieve one of the rings of the SRR. The electrical behavior of this structure will be studied as a function of temperature. The outcome of this experiment is essential for the further construction of the metamaterial, because the electrical properties of the metallic structures determine the optical properties later achievable in ordered arrays of these nanostructures. These tests need to be performed in order to select suitable candidates for the construction of the metamaterial.

This fabrication scheme of DNA templated metallic nanostructures and their electrical characterization are examples for the combination of “top-down” and “bottom-up” approaches in nanotechnology. The DNA Origami method and the functionalization with NPs to generate structures at the nanoscale are bottom-up approaches. The lithographic methods used for contacting the resulting C-shape rings are top-down methods.

**Figure 1:**
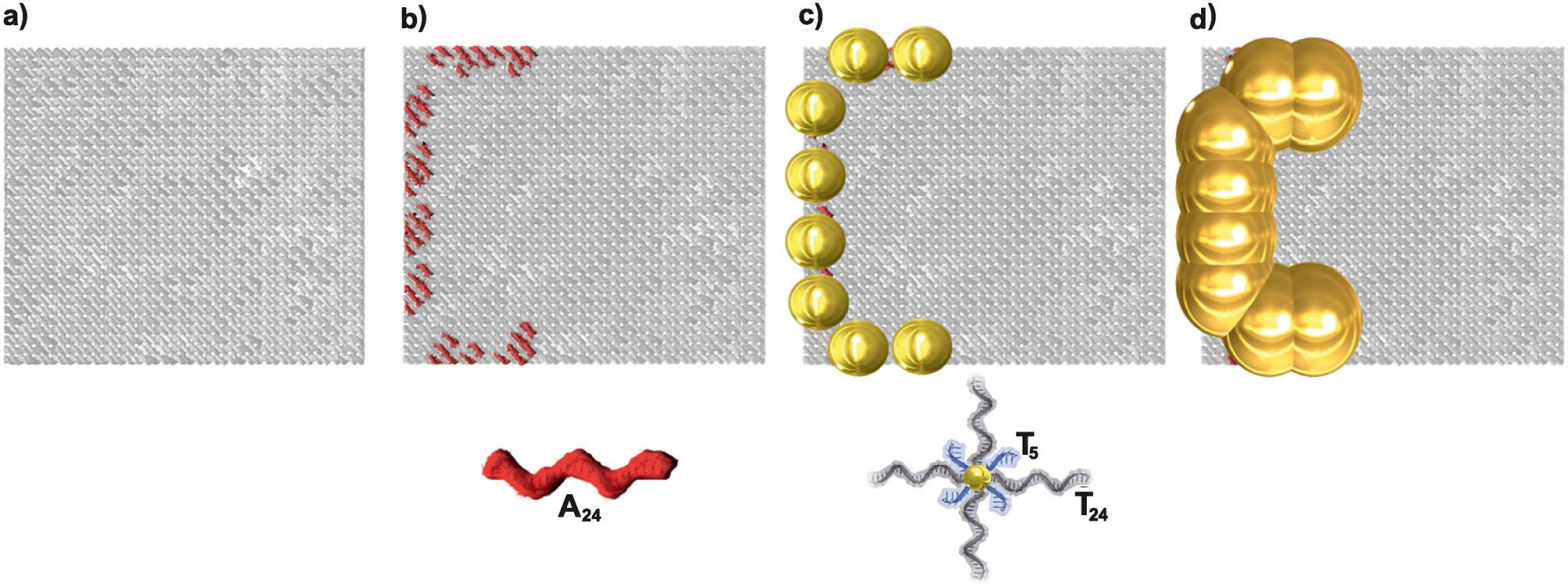
Schematic illustration showing the construction of C-shape wires based on DNA origami nanosheets. (**a**) Scheme of the DNA origami nanosheet, ${90}\hbox { nm}\times {70}\hbox { nm}$, as a template for the assembly of DNA functionalized AuNPs. (**b**) Three sides of the DNA nanosheet are functionalized with sequences consisting of 24 adenines ($A_{24}$) for binding 8 AuNPs. (**c**) AuNPs, ${5}\hbox { nm}$ in diameter, functionalized with thymine DNA single strands ($T_{24}$) and complementary to the $A_{24}$ on the DNA origami nanosheet, are attached on specific locations by hybridization. The thymines are followed by a 5 base spacer ($T_5$) for increased flexibility. (**d**) Electroless gold deposition on the AuNPs results in formation of DNA nanosheet templated gold wires with a C-shape.

The electrical characterization of nanowires was done using electron beam lithography (EBL)^19,22,23,27–29^ with electrodes fabricated to the ends of the C-shape wires^12,20,30–32^. Electric charge transport of such wires shows a widespread range in resistances from a few tens of ohms up to several gigaohms. These results suggest that the fashioning of metal NPs on the origami nanostructure and their coalescence during metallization control the resistance, whereas the length and width of the final structure hardly influence the electrical properties^19,27,28,33^. Temperature-dependent electrical characterization of nanowires generated by DNA metallization has shown that these wires are not completely continuous^33^. Merely, they often contain gaps between the metal nanoparticles which appear during growing and merging the AuNPs on the surface of the DNA origami^34^

Here, we analyze the electric transport of C-shape gold nanowires based on small DNA origami templates (${90}\hbox { nm} \times {70}\hbox { nm}$ DNA origami nanosheets) having three functionalized sides, holding eight gold nanoparticles conforming one of the rings of a SRR (see Fig. 1). The DNA origami nanosheet design is shown in figure S1 and the staple strand sequences are given in tables S1 and S2 in the supporting information (SI). Electroless gold growth is applied to selectively grow the gold nanoparticles until they merge into nanowires. Finally, this work demonstrates the fabrication of single and isolated conductive nanowires with a C-shape. They are precisely contacted by metal using EBL to understand the charge transport characteristics at different temperatures. In particular, we need to answer the following questions:

1. Can we create nanowires with specific shapes (in this case C-shape with a length of 150 nm) with good reliability using chosen DNA origami template?
2. Are the electrical properties of the nanostructures suitable for the application for which they have been designed?

Specifically, the C-shape was designed for generating a metamaterial for optical applications. For such applications the electrical conductivity is important, because it determines the ability to couple electromagnetic waves. Optical characterization of the metamaterials is not possible before ordered deposition of the origami structures can be achieved. It is, however, necessary to characterize the nanostructures before the development of the deposition, because the deposition depends on the properties of the nanostructures, as well. Therefore, the first step in the construction of a metamaterial is the testing of the electrical properties. In order to proceed with the metamaterial production, it is not only essential to have single structures with good properties, but these structures are also needed with good statistics. We therefore investigate the statistical properties of the binding of the nanoparticles and the distribution of their electronic properties. To the best of our knowledge, no such study has been performed up to date.

## Results and Discussion

**Figure 2:**
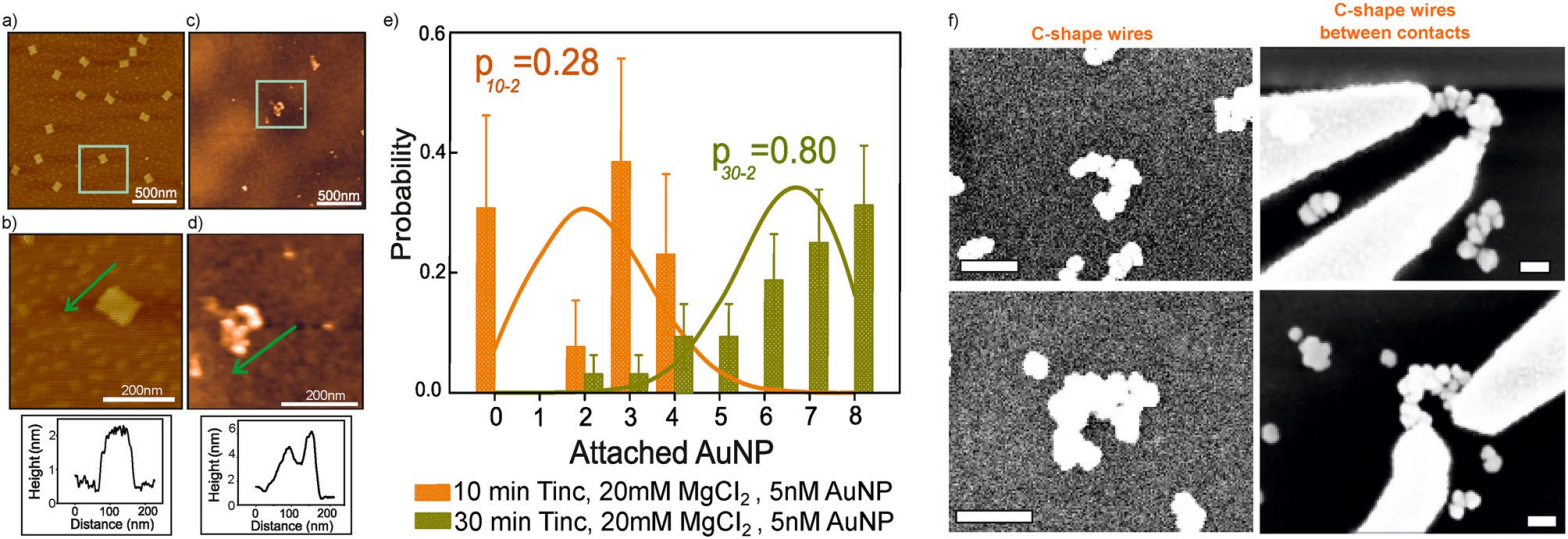
(**a**) AFM height images of immobilized bare DNA origami sheets and (**b**) a high magnification AFM image of an individual nanosheet (green box in **a**)) on a $\mathrm {SiO_2}$ surface. (**c**) AuNPs attached to DNA origami at specific positions with high attachment yield and (**d**) high magnification AFM image of an individual AuNP decorated nanosheet (green box in **c**)). Inset figures show a cross-sectional profile of the individual origami and AuNPs decorated origami in b) and d), respectively. Green arrows indicate scan direction. (**e**) Measured attachment probabilities of AuNPs obtained from collected AFM images (histograms) and expected binomial distributions (solid lines) for two experimental conditions. The average attachment probabilities of AuNPs site occupation, p, are given for each case. (**f**) SEM images of two typical C-shape nanowires before and after being contacted by Au electrodes. Scale bars in in SEM images are ${250}\hbox { nm}$ and ${100}\hbox { nm}$ before and after gold contacts, respectively.

Figure 1 shows a schematic representation of the different stages to build the C-shape nanowires from the assembly of eight AuNPs on a rectangular DNA origami, or nanosheet. The nanoparticles have a diameter of ${5}\hbox { nm}$, and their mutual distance can be programmed by the design of the origami. The DNA origami nanosheets are designed to have a size of ${90}\hbox { nm}\times {70}\hbox { nm}$, as shown in Fig. 1a. For the attachment of the AuNPs, a total of eight pairs of staple strands have been extended at specific positions on each DNA origami nanosheet as displayed in Fig. 1b. They were designed as capture strands through their $5'$ end to create eight binding sites for capturing AuNPs, see Fig. 1c and SI-figure S2. The design of the binding sites establishes the distance from center-to-center of two neighboring AuNP binding sites to ${16}\hbox { nm}$ to form a C-shape nanowire after metallization, as shown in Fig. 1d. Figure 2a–d show low- and high-magnification atomic force microscopy (AFM) height images and height profiles of the origami nanosheets placed on $\mathrm {SiO_2}$/Si substrates before and after the attachment of AuNPs. The mean length and width of the rectangular nanosheet was measured to be 90 $\pm ~{2}\hbox { nm}$ and 70 $\pm ~{2}\hbox { nm}$ , respectively, for over 100 samples. As seen from the inset in Fig. 2d, the average height of DNA origami with attached AuNPs was   5 $\pm ~{0.5}\hbox { nm}$.

The yield of AuNP attachment, i.e. seed placement, is highly dependent on the stability and adherence of the DNA origami templates deposited on $\mathrm {SiO_2}$/Si. Due to mechanical instabilities and deformations, the templates tend to bend in such a way that the unfunctionalized side covers those sides (see SI- Figure S3c) with the capture strands hindering the AuNP attachment during the hybridization process. To reach a high yield of base-pairing between binding sites of origami and functionalized AuNPs, the $\mathrm {Mg^{2+}}$ concentration in the buffer, AuNP concentration and functionalization times ($T_ {\rm inc}$) have been varied. This AuNP attachment process was optimized by carrying out several tests on dried samples at room temperature (RT) in $1\times$ TAE buffer (Solution containing a mixture of Tris base, Acetic acid and Ethylenediamine tetraacetic acid (EDTA)) having different $\mathrm {Mg^{+2}}$ concentrations (20, 50 mM magnesium chloride ($\mathrm {MgCI_2}$)). In these tests, incubation times of 10, 20, and ${30}\hbox { min}$, and molar concentration ratios at 2.5:1 and 5:1 of functionalized AuNPs to DNA origami nanosheets were chosen. The statistical analysis of the number of AuNPs attached to the DNA origami with fixed binding sites under the experimental conditions given above is shown as a series of histograms in the SI-figure S4a–d. The data in the histograms were collected from AFM images for over 900 separate origami nanosheets of several samples.

The histograms show that a long incubation time, ${30}\hbox { min}$, a concentration of 20 mM ${\hbox {MgCI}_2}$ in the buffer and a low molar ratio (2.5:1) of functionalized AuNPs to DNA origami lead to the highest probability for attaching 8 AuNPs at the respective binding sites. The average attachment probability of AuNP site occupation, *p*, is defined by^35^:

1 $$\begin{aligned} p = \frac{\Sigma \text {(attached NPs)}}{\Sigma \text {(available sites)}} \end{aligned}$$

Deviations from the ideal case, *i.e. *binding of 8 particles to the respective binding sites on the origami, are known to be caused by: (i) physical crowding due to neighbouring nanoparticles (steric effects)^36^, (ii) Coulomb interactions of nanoparticles (electrostatic repulsion), and (iii) individual nanoparticles bridging various sites (site bridging)^35,37–40^. Disregarding these interactions, AuNP attachments are considered as independent and the histograms are predicted to be binomial distributions, *p*(*m*), given by^14,35^:

2 $$\begin{aligned} p(m) = \frac{n!}{m!\,(n-m)!} p^m (1-p)^{(n-m)} \end{aligned}$$

where *n*, *m* and *p* are the number of available binding sites, number of AuNPs attached per origami nanosheet, and average probability of AuNPs site occupation, respectively. These values were obtained from the histograms of the number of attached AuNPs per nanosheet, which have been compiled from the AFM data, to accomplish the binomial distribution for each experimental condition. The results of the statistical analysis are given in Table S3 in the SI. The solid lines in the histograms in Fig. 2e and SI-figure S4 show the calculated binomial distribution following equation (2). It can be clearly seen that for a high attachment probability, the experimentally achieved distribution deviates from the binomial distribution. This is an indication that at these high attachment probabilities steric effects and/or Coulomb interactions between the nanoparticles may play an important role in determining the yield of successful binding events.

**Figure 3:**
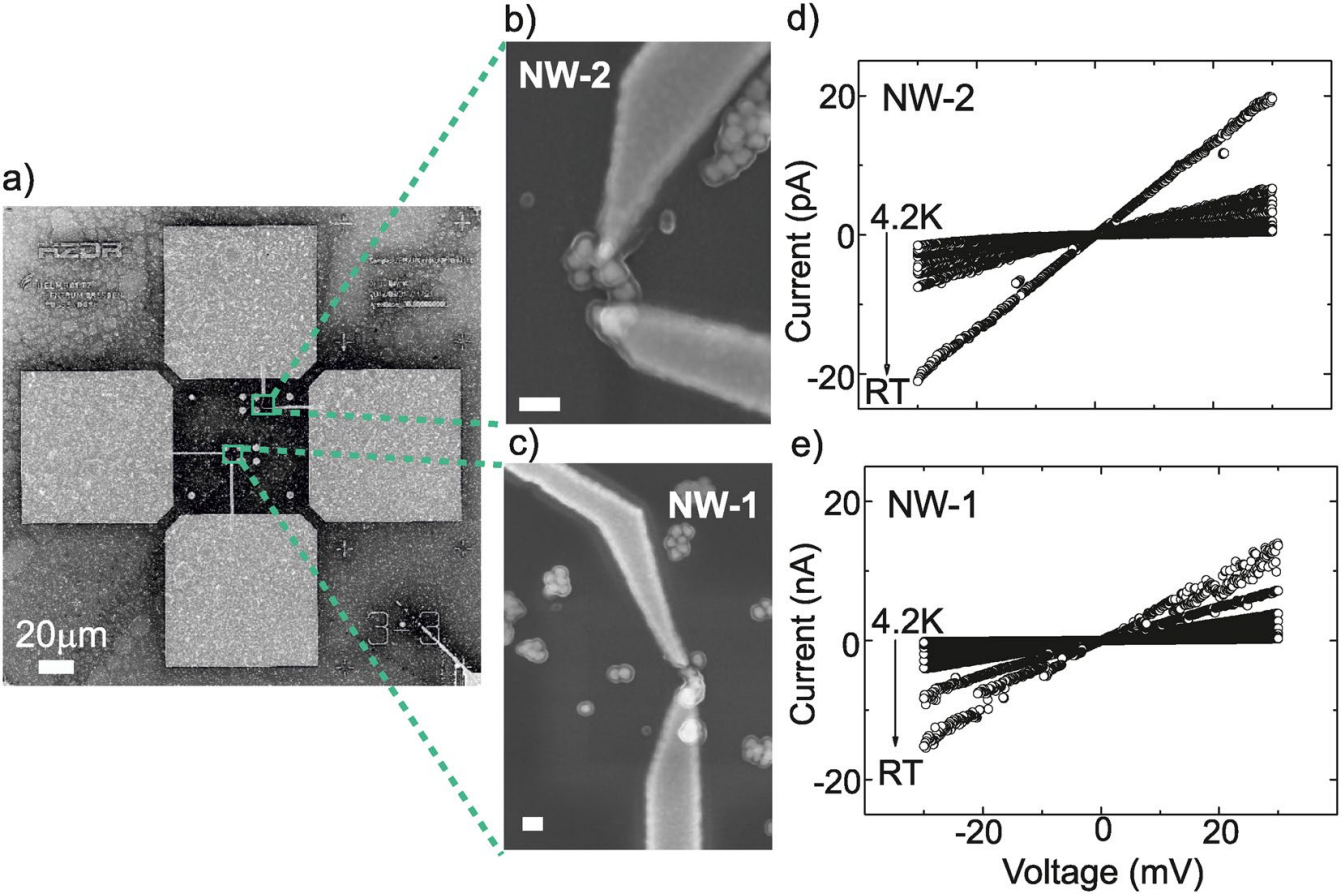
(**a**) SEM images of electrical contact pads and markers on ${\hbox {SiO}_2 / \hbox {Si}}$ substrates. The markers are laid out on a square lattcie which define the “writing” fields (green squares) of ${8}\,\upmu \hbox {m}$ x ${8}\,\upmu \hbox {m}$. (**b**), (**c**) Individual C-shape nanowires in between source and drain electrodes. Scale bars are ${100}\hbox { nm}$. (**d**), (**e**) Temperature dependent *I*-*V* characteristics from 4.2 K to RT of C-shape wires by biasing from ${-30} \hbox { mV}$ to ${-30} \hbox { mV}$. The x-axis labels are identical for (**d**), (**e**).

In particular, the average attachment probability was calculated to be 0.80 for $T_ {\rm inc}$ = 30 min, a concentration of 20 mM $\mathrm {MgCl_2}$ and 2.5:1 molar ratio of functionalized AuNPs to DNA origami, as seen from Table S3. The highest AuNPs site occupation probability reported in literature is $p=0.99$ for a DNA origami nanorail ($6\times 2$ helix bundle) with base-pairing interaction for four capture strands per binding site^35^. The reduction of the attachment probability is most likely caused by the geometry of the DNA origami and, related to this, by the number of available capture strands per particle. The geometry, however, is crucial for the application of the origami technique in nanoelectronics. Therefore, the attachment probability of 80% is used for the electrical characterization of these proof-of-concept devices discussed here.

The C-shape gold nanowires were afterwards formed by enhancement of the AuNPs using a mixture of gold plating solution and 20 mM $\hbox {MgCl}_{2}$ in a $1 \times$ TAE buffer for 1:1 and 1:2 volume combinations. Each of these mixtures was dripped onto the AuNP:DNA origami conjugates for different incubation times, 2, 7, 14 and 20 min. At a 1:1 volume ratio, an instantaneous coalescence of gold was observed on the AuNPs, which act as nucleation centers. No control on AuNP growth was possible for a 1:1 ratio. However, as observed by AFM, the ${5}\hbox { nm}$ AuNPs grew up to ${8}\hbox { nm}$ without any mergence between them after 2 min of incubation time for the 1:2 mixture. The AuNPs grew even further up to  ${10.5}\hbox { nm}$ and  ${14}\hbox { nm}$ when the incubation time was 7 and ${14}~\hbox {min}$ , respectively. Finally, the AuNPs merged to form C-shape nanowires after an incubation time of ${20}~\hbox {min}$ but exhibited sporadic tiny gaps along the wires analyzed by scanning electron microscopy (SEM), as shown in Fig. 2f.

Two-terminal current–voltage (*I*–*V*) measurements were completed on 16 individual wires using a contact geometry as shown in Fig. 3. The measured resistance values at RT in dry conditions including the contact resistance of gold electrodes and C-shape wires were between ${2.3}~{\hbox {M}\Omega }$ and ${250}~{\hbox {G}\Omega }$, being far lower than those measured for unfunctionalized DNA origami nanosheets with similar dimensions^41^. SEM images and *I*–*V* curves of contacted C-shape wires are given in SI–figures S6 and S7, a comparison of the resistance values of these wires is given in SI–figure S8. Two out of 16 contacted nanowires in Fig. 3b, c were chosen to determine the dependence on temperature of the charge transport mechanism. For ease of discussion, the nanowires are referred to as NW-1 and NW-2. Figure 3b,c show magnified SEM images of Fig. 3a for NW-1 and NW-2 having two electrodes on each wire with corresponding gold-gold contacts.

**Figure 4:**
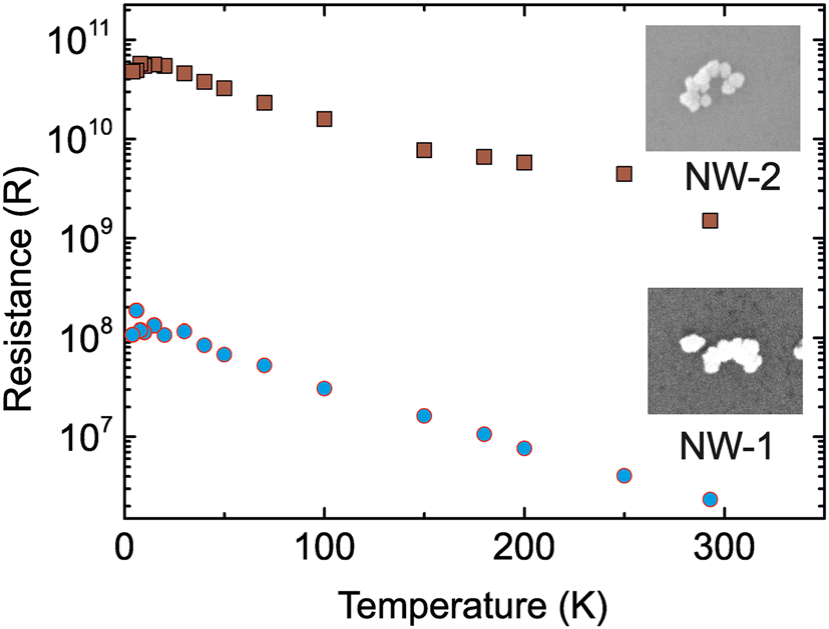
Resistance versus temperature curves for two Au-metallized C-shape nanowires, named NW-1 and NW-2, templated by DNA origami in the 4.2 K to RT temperature range. Scanning electron microscopy images of the two nanowires can be seen as inset figures.

Temperature-dependent *I*–*V* characterization was performed in the voltage range between -30 and 30 mV. Figure 3d,e show that both metallized C-shape nanowires have an ohmic behavior at RT (293 K), and the resistances were found to be ${2.3}~{\hbox {M}\Omega }$ and ${1.4}~{\hbox {G}\Omega }$ for NW-1 and NW-2, respectively. In order to understand the nature of the transport process through each wire, *I*–*V* sweeps were performed at different temperatures from 4.2 K to RT in the voltage range mentioned above; these data are shown in Fig. 3d,e. It is observed that the resistance in the linear regime $R=\left( \frac{dI}{dV}\vert _{V=0}\right) ^{-1}$ increases from ${2.3}~{\hbox {M}\Omega }$ (RT) to ${107}~{\hbox {M}\Omega }$ (4.2K) for NW-1 and ${1.4}~{\hbox {G}\Omega }$ (RT) to ${48}~{\hbox {G}\Omega }$ (4.2 K) for NW-2, as shown in Fig. 4. The magnified SEM images in the inset figures in figure 4 demonstrate that NW-1 and NW-2 have different morphologies. In particular, NW-1 has densely coalesced AuNPs due to fine metallization; in contrast, NW-2 has coarse grain boundaries between merged NPs. These structural dissimilarities between NW-1 and NW-2 might explain the resistance differences shown in Fig. 4.

The high resistance values are mainly caused by imperfections in the metallization of the wires, which cause gaps between the nanoparticles even after growth. These gaps can be clearly seen in the high magnification SEM images in Fig. 4.

**Figure 5:**
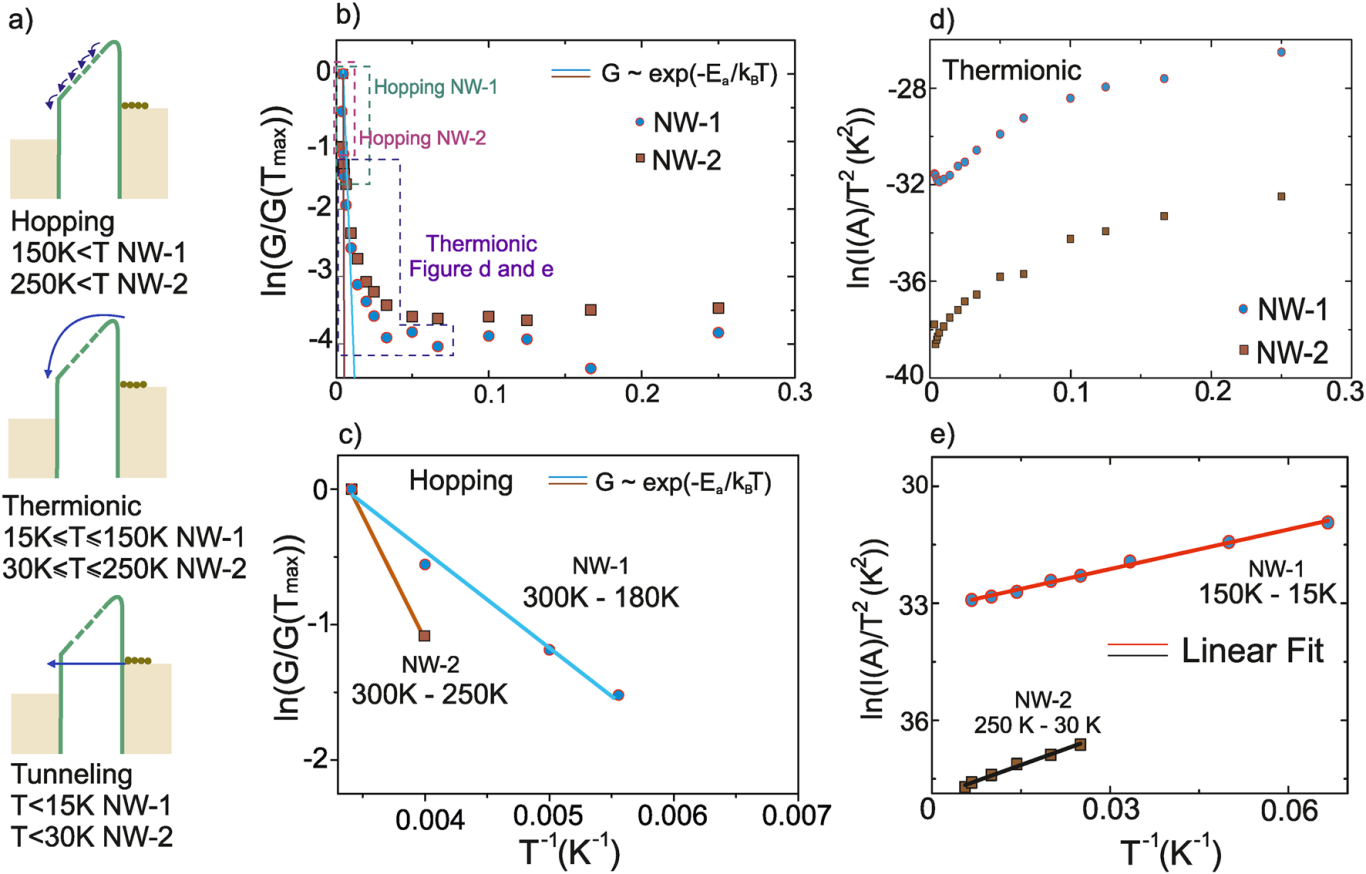
(**a**) Representative diagram of hopping, thermionic and tunneling injection processes over the barrier between metallic contacts and nanoparticles. (**b**) Inverse temperature dependence of conductance for NW-1 and NW-2. (**c**) Linear fit of data shown in (**b**) in the high-temperature range, in which hopping is the dominant charge transport mechanism. (**d**) Plot of $ln(I)/T^2$ according to the Richardson-Schottky model describing thermionic emission. (**e**) Linear fit of the data shown in d) in the intermediate temperature range, in which thermionic emission dominates charge transport.

Based on the electrical measurements, Fig. 5a shows a simple band model for electron transport through C-shape gold nanowires for the different temperature regimes for NW-1 and NW-2. In Fig. 5b, the logarithm of the normalized conductance $\left( \ln \frac{G}{G(T_ {\rm max})}\right)$ has been plotted as a function of the reciprocal temperature ($T^{-1}$). It is observed that the conductance is limited by the activation energy $E_a$ in the electron transport through NW-1 and NW-2 in the high temperature range. The activation energies of the wires were acquired from the exponential part of the conductance based on the fit function $G \sim \exp \frac{-E_a}{k_B T}$, where $k_B$ is the Boltzmann constant. At “high” temperatures (180 K to RT for NW-1 and 250 K to RT for NW-2) and low voltage bias ($\le {30}\hbox { mV}$), the conductance strongly depends on temperature which results in values for $E_a$ of 60 meV and 160 meV for NW-1 and NW-2, respectively, as shown in Fig. 5c. Charge transport is dominated by hopping at this temperature range. At intermediate temperatures, thermionic conduction governs the charge transport which is associated with the Richardson-Schottky equation for thermionic emission^42^:

3 $$\begin{aligned} I \sim A^* \cdot T^2 \exp \left( \frac{{\Phi } - {(\frac{q^3 V}{4 \pi \epsilon d}})^{1/2}}{k_B T}\right) \end{aligned}$$

where $\Phi$ is the barrier height, $A^*$ is the effective Richardson constant, $\epsilon$ and *d* are the permittivity of vacuum and the barrier width, respectively. Figure 5d shows graphs of $\ln \left( \frac{I}{T^2}\right)$ as a function of ${T^{-1}}$ for both wires in the temperature range from 4.2 K to RT. For both wires, this plot shows a linear region, in which thermionic emission is most likely dominating. The temperature range in which this holds true depends on the morphology of the wire. In our measurements, it extends from 15 K to 150 K and from 30 K to 250 K for NW-1 and NW-2, respectively, as shown in Fig. 5e.

Direct tunneling of the electrons through an energy barrier governs the electrical conductance at very low temperatures, from 4.2 K to 15 K for NW-1 and from 4.2 K to 30 K for NW-2. In these lowest temperature ranges, the measured conductance is independent of temperature. The resistance of the most insulating wire (${15}~{\hbox {G}\Omega }$) was measured again at increasing applied voltage bias values of ${30} \hbox { mV}$, ${50}\hbox { mV}$, ${1} \hbox { V}$, ${2} \hbox { V}$, ${3} \hbox { V}$ and ${5} \hbox { V}$ then back to 30 mv. Afterwards, the measured resistance decreased to ${4}~{\hbox {G}\Omega }$ (See SI-Figure S5). The voltage-induced electric fields probably reduced the gaps along the wire which normally occurs during thermal annealing or electro-migration^43^.

## Methods

The previously reported “tall rectangle” DNA origami^44^ (${90}\hbox { nm}\times {70}\hbox { nm}$) was used as the DNA origami nanosheet template for the metallic structure presented here. The origami was formed by folding an M13mp18 bacteriophage single-strand DNA using more than 200 synthetic oligonucleotide staple strands, as originally designed by Rothemund^2^ but with the following changes: Along the perimeter of the rectangle, some of the side staples were elongated by means of 5 adenines ($A_5$) to prevent aggregation *via* helix stacking. Additionally, two consecutive staples were located at each of the AuNPs binding sites for placing eight NPs in total to produce a C-shape array. These staples were extended on the $3'$ end followed by a specific 24 adenine sequence ($A_{24}$) for each site. One of these $A_{24}$ extensions was in the $5'\rightarrow 3'$ direction while the adjacent one was in the $3'\rightarrow 5'$ direction, so they do not contend each other during AuNP functionalization, see SI–figure S2. These extended staples were chosen so that the extensions would be oriented perpendicular, away from the origami, as shown in Fig. 1 b).

The synthesis of the DNA origami nanostructures was performed by first pipetting ${2}~\upmu \hbox {l}$ from a ${0.4}~\upmu \hbox {M}$ M13mp18 ssDNA solution, and then adding ${10}~\upmu \hbox {l}$ of a ${0.44}~\upmu \hbox {M}$ mix of single strand staples. Secondly, ${12}~\upmu \hbox {l}$ $10\times$TAE/$\mathrm {Mg^{2+}}$ and ${96}~\upmu \hbox {l}$ de-ionized (DI) water were mixed to the previous solution. Finally, this mixture was annealed in a programmed thermal cycler from ${94}\,^\circ \hbox {c}$ to ${4}\,^\circ \hbox {c}$ with a temperature ramp of ${1}\,^{\circ} \hbox {C}/\hbox {min}$ and stored at ${4}\,^\circ \hbox {c}$. Afterwards, the prepared origami (5 nm) was filtered by performing four buffer exchanges in $1\times$TAE/12.5 mM $\mathrm {Mg^{2+}}$ buffer (40 mM Tris-acetate, 1 mM EDTA, pH 8.2) with a single ultra-filtration centrifuge unit (100kDa MWCO, Millipore) to remove the extra staple strands.

The functionalized AuNPs which are attached at the specific binding sites to the “tall rectangle” origami were prepared as follows. The AuNPs (British Biocell International) of 5 nm in size were conjugated to a specific oligonucleotide chain of 24 thymines ($T_{24}$), complementary to the 24 adenines ($A_{24}$) described above, at an AuNP:ssDNA ratio of 2.5:1 and allowed to incubate overnight. The $T_{24}$ capture single strands had a disulfide modification on either the 5’ end or 3’ end to be hybridized on the $A_{24}$ extensions either in the direction $3' \rightarrow 5'$ or $5'\rightarrow 3'$, respectively, and followed by a 5 base spacer (T5) for increased flexibility. After overnight incubation, the AuNPs were then backfilled with thiolated $T_5$ sequences to prevent AuNPs aggregation in presence of high $\mathrm {Mg^{2+}}$ concentration buffers and left incubating for another 24-h period. The conjugated AuNPs:ssDNA were ready to be used.

The rectangular shape DNA origami nanosheets were deposited on p-Si (100) with a ${300}\hbox { nm}$ $\mathrm {SiO_2}$ electrical insulation layer. Before dropping these nanosheets, the wafer pieces were pre-treated in an $\mathrm {O_2}$ plasma (PICO, Diener Electronic-Plasma Surface Technology) at 7sccm $\mathrm {O_2}$, power of 240 W for ${3}~\hbox {min}$ to render their surfaces hydrophilic. At that moment every sample holder was rinsed with ethanol (20s), DI water (20s) and dried in a $\mathrm {N_2}$ stream. The concentration of the nanosheets was then diluted to 2 nm and a volume of ${20}~\upmu \hbox {l}$ of this solution was dropped on the sample holder surface immediately after drying and incubated for 1h in $10\times$TAE/200 mM $\mathrm {Mg^{2+}}$ in a humidity chamber.

The metallization of the DNA origami nanosheets in order to create C-shape Au nanowires consists of two steps: (i) hybridization of AuNPs:ssDNA on the DNA origami nanosheets and (ii) enhancement of AuNPs by reduction using an Au plating solution kit (GoldEnhance EM, Nanoprobes). Firstly, to control nanoparticle assembly and reach high attachment yield, hybridization of AuNPs onto the immobilized DNA origami nanosheet was performed by incubation at 2.5:1 or 5:1 molar ratios of functionalized AuNPs to DNA nanosheets for 10, 20 and 30 min at room temperature in a 1 $\times$ TAE buffer containing 20 or 50 mM $\mathrm {MgCl_2}$. The residual salt was cleaned using ${1000}~\upmu \hbox {L}$ of 1:1 mixture of ethanol:Milli-Q water to remove $\mathrm {Mg^{2+}}$ residue from the surface. Excess water was blown off in a $\mathrm {N_2}$ stream, but the origamis were kept in a humidity chamber to keep the origamis from drying out.

Subsequently, AuNPs were enhanced to homogeneous C-shape metallic nanowires. The growth of the AuNPs was controlled by means of 1:1 and 1:2 mixtures of gold plating solution and 20 mM $\mathrm {MgCl_2}$ in a 1 $\times$ TAE buffer. These mixtures were dropped onto the AuNP:DNA origami conjugates with different incubation times, 2, 7, 14 and 20 min. Rectangular DNA origami structures on $\mathrm {SiO_2/Si}$ substrates and AuNP assemblies were characterized by AFM operating in tapping mode using a Bruker MultiMode 8 Scanning Probe Microscope and aluminum reflex coated tips (Tap150Al-G from Nanoandmore, force constant ${5} \hbox {Nm}^{-1}$, tip radius $<{10}\hbox { nm}$). The topographic AFM images were analyzed using both the Gwyddion software and the one provided with the Bruker instrument. C-shape metallized DNA origamis were also analyzed by SEM with a 30 mM aperture size at 10 keV beam energy.

The electrical contact pads and markers were fabricated using electron beam lithography (Raith e-line Plus) on the $\mathrm {SiO_2}$ surface on which DNA origami-based wires have been deposited in advance. ZEP250 electron beam resist was spin coated and baked at ${150}\,^\circ \hbox {C}$ for ${10}~\hbox {min}$. The resist was exposed at 10 kV acceleration voltage with aperture sizes of ${15}\upmu \hbox {M}$ and ${7}\,\upmu \hbox {M}$ to ${10}\,\upmu \hbox {M}$ for contacts and markers, respectively. The resist was developed in N-amyl acetate for 90s and then in isopropanol (IPA) for 30 s. A ${5}\hbox { nm}$ titanium adhesion layer followed by ${70}\hbox { nm}$ gold were evaporated at 0.2Å/s and 2Å/s rates, respectively, by e-beam evaporation (Bestec Evaporation Tool). The lift-off process was performed by immersion in ZDMAC (Dimethylacetamide) and subsequent washing with IPA, followed by $\mathrm {N_2}$ drying.

In order to contact individual C-shape nanowires for electrical transport measurements, sets of alignment marks in parallel arrays with ${8}\,\upmu \hbox {M}$ gaps in between were patterned by means of electron beam lithography on $\mathrm {SiO_2/Si}$ surface, as seen in Fig. 3a, using experimental conditions as previously reported^19^. The relevant positions of the individual wires were determined relative to the alignment marks using SEM. These locations were used to obtain corresponding e-beam exposure positions in order to place electrical contacts on the nanowires. SEM images confirmed that the DNA origami templated C-shape wires are between electrical contacts with high accuracy.

Two-terminal current–voltage (*I*–*V*) measurements were completed on 16 individual wires, shown in Fig. 2f and SI-S6 and S7 before and after Au contacts. Electrical measurements were carried out using a semiconductor parameter analyzer (Agilent 4156-C) in vacuum (${10^{-5}}$ mbar base pressure) and in darkness using two probes. To measure the current-voltage characteristics of wires between electrodes, tungsten tips (25 mm) were placed on the gold contact pads. A liquid helium continuous flow cryostat system was used for temperature-dependent electrical measurements. The samples were cooled down to 4.2 K and measurements were performed while heating up (${10^{-8}}$ mbar working pressure). *I*-*V* measurements were done by sweeping the voltage from 0 to 30 mV and from 0 to -30 mV. The resistance of the nanowires was determined through a linear fit to the measured *I*-*V* curves. For wires which showed non-linear *I*-*V* behavior at low temperatures, the zero-bias resistance was determined as the slope of the curve at the inflection point near zero voltage.

## Conclusions

We have demonstrated that C-shape Au-nanowires with an overall length of ${150}\hbox { nm}$ could be self-assembled using a rectangular DNA origami (${90}\hbox { nm} \times {70}\hbox { nm}$) as a template. The attachment probability of the Au-nanoparticles constituting the nanowires was optimized for the desired patterns. The electrical properties of these nanowires were tested at temperatures ranging from 4.2 K to RT (293 K).

This is the first step in the construction of a split-ring resonator using a combination of bottom-up and top-down methods. The particular shape of the nanowires was obtained by the binding of 8 functionalized AuNPs at specific capture sites for each and strategically separated by ${16}\hbox { nm}$ on the DNA origami surface. The AuNPs are subsequently grown up to mergence by means of an enhancement solution. The resulting shapes of the nanowires show that the strategy of using nanoparticles for the definition of the curved wires leads indeed to the desired c-shape. These findings demonstrate that the nanowires, despite their reduced dimensions and curved shape, are as good in electrical quality as those previously manufactured, longer straight wires^27,34^ assembled with similar strategies. The success in the production of gold nanowires having a low resistance with ohmic behavior strongly depends on the several stages of the nanowire synthesis. Therefore, future studies will concentrate on the improvement of mechanical stability and adherence of the DNA origami serving as a template for the efficient assembly of the AuNPs. First demonstration of high-quality metallic wires have been produced by controlling and confining the metal growth within three dimensional DNA origami mold structures^19,34^. This approach indicates a reliable route for fabricating continuous and smooth metallic nanostructures with well-defined morphology using DNA nanotechnology.

## Supplementary Information

Supplementary Information
