# Supplementary material for: Fabrication and temperature-dependent electrical characterization of a C-shape nanowire patterned by a DNA origami
Source: Sci Rep. 2021 Jan 21;11:1922. doi: 10.1038/s41598-021-81178-8 (PMC7820232; doi:10.1038/s41598-021-81178-8)
Supplement: Supplementary file 1 — Supplementary Information [file 41598_2021_81178_MOESM1_ESM.pdf]

Supporting Information

Fabrication and temperature-dependent electrical characterization of a C-shape nanowire patterned by a DNA origami

Turkan Bayrak[1,2] , Amanda Martinez-Reyes[3], David Daniel Ruiz Arce[3], Jeffrey Kelling[4], Enrique C. Samano[3,*] and Artur Erbe[1,2,*]

[1] Institute of Ion Beam Physics and Materials Research, Helmholtz-Zentrum Dresden-Rossendorf, 01328 Dresden, Germany.

[2] Cluster of Excellence Center for Advancing Electronics Dresden (cfaed), TU Dresden, 01062 Dresden, Germany.

[3] Centro de Nanociencias y Nanotecnología, Universidad Nacional Autónoma de México, Ensenada, B.C., México.

[4] Institute of Computational Science, Helmholtz-Zentrum Dresden-Rossendorf, 01328 Dresden, Germany.

* Correspondence should be addressed to Enrique C. Samano (samano@cnyn.unam.mx) or Artur Erbe (a.erbe@hzdr.de, Tel. +49 351 260 2366)

All DNA origami staples, including modified staples, and complementary single strands attached to the AuNPs were purchased from Integrated DNA Technologies (IDT), Inc. The purity of all oligonucleotides used in this work is the standard desalting kind that provides IDT and all the staples are LabReady (100 μM in IDTE, pH 8.0).

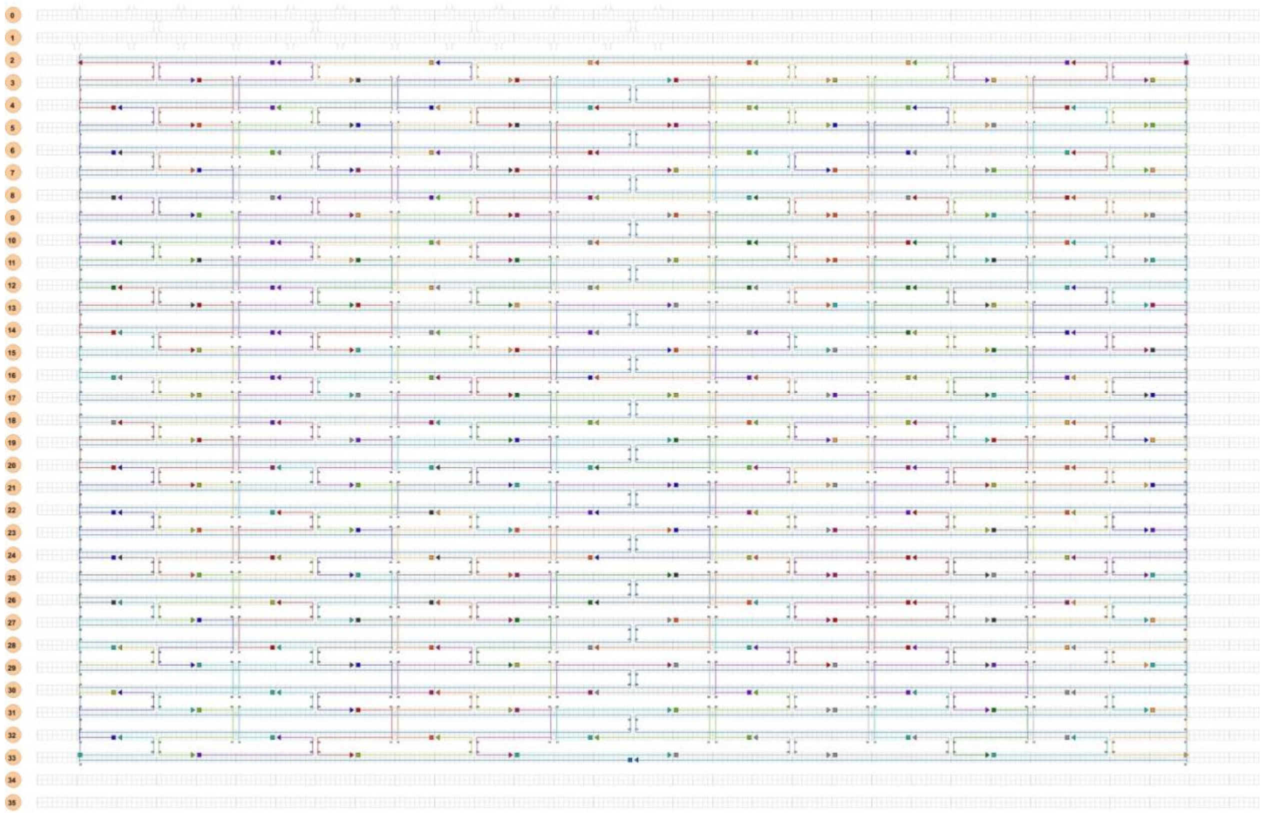

Figure S1. Schematic diagram of the DNA origami nanosheet sequences. The single strand scaffold, the M13mp18 genome, and the complementary staples are shown by fonts in black and different colors, respectively. The specific length and sequences of the 225 staple strands are listed in the table S1.

Sequences for the tall rectangle (bridged seam)

Table S1. Staple strand sequences for the normal tall rectangle origami, also called nanosheet.
.

S.No.	Sequence code	Sequence (5'-3')
1.	t1r0g	AGGGTTGATATAAGTATAGCCCGGAATAGGTG
2.	t1r10e	TGAACAAAGATAACCCACAAGAATAAGACTCC
3.	t1r10f	ATCAGAGAGTCAGAGGGTAATTGAACCAGTCA
4.	t1r12e	TATTTTGCACGCTAACGAGCGTCTGAACACCC
5.	t1r12f	TCTTACCAACCCAGCTACAATTTTAAAGAAGT
6.	t1r14e	ATCGGCTGACCAAGTACCGCACTCTTAGTTGC
7.	t1r14f	GGTATTAATCTTTCCTTATCATTCATATCGCG
8.	t1r16e	CATATTTATTTCGAGCCAGTAATAAATCAATA
9.	t1r16f	AGAGGCATACAACGCCAACATGTATCTGCGAA
10.	t1r18e	ACAAAGAAAATTTCATCTTCTGACAGAATCGC
11.	t1r18f	TTTTAGTTCGCGAGAAAACTTTTTTTATGACC
12.	t1r20e	AAATCAATCGTCGCTATTAATTAAATCGCAAG
13.	t1r20f	CTGTAAATATATGTGAGTGAATAAAAAGGCTA
14.	t1r22e	TTTAACGTTCGGGAGAAACAATAACAGTACAT
15.	t1r22f	CTTTTACACAGATGAATATACAGTGCCATCAA
16.	t1r24e	TTATTAATGAACAAAGAAACCACCTTTTCAGG
17.	t1r24f	ATTTTGCGTTTAAAAGTTTGAGTACCGGCACC
18.	t1r26e	CTAAAGCAAATCAATATCTGGTCACCCGAACG
19.	t1r26f	AAACCCTCTCACCTTGCTGAACCTAGAGGATC
20.	t1r28e	GCCAACAGATACGTGGCACAGACATGAAAAAT
21.	t1r28f	GCGTAAGAAGATAGAACCCTTCTGAACGCGCG

22.	t1r2e	TAAGCGTCGGTAATAAGTTTTAACCCGTCGAG
23.	t1r2f	AGTGTACTATACATGGCTTTTGATCTTTCCAG
24.	t1r30e	GTTGTAGCCCTGAGTAGAAGAACTACATTCTG
25.	t1r30f	ATCACTTGAATACTTCTTTGATTAGTTGTTCC
26.	t1r32h	TACAGGGCGCGTACTATGGTTGCTAATTAACC
27.	t1r4e	AACCAGAGACCCTCAGAACCGCCACGTTCCAG
28.	t1r4f	GAGCCGCCCCACCACCGGAACCGCTGCGCCGA
29.	t1r6e	GACTTGAGGTAGCACCATTACCATATCACCGG
30.	t1r6f	AATCACCACCATTTGGGAATTAGACCAACCTA
31.	t1r8e	TTATTACGTAAAGGTGGCAACATACCGTCACC
32.	t1r8f	TACATACACAGTATGTTAGCAAACTGTACAGA
33.	t3r0g	TGCTCAGTACCAGGCGGATAAGTGGGGGTCAG
34.	t3r10e	GCGCATTAATAAGAGCAAGAAACAATAACGGA
35.	t3r10f	GCCCAATAGACGGGAGAATTAACTTTCCAGAG
36.	t3r12e	AGGTTTTGGCCAGTTACAAAATAAACAGGGAA
37.	t3r12f	CCTAATTTAAGCCTTAAATCAAGAATCGAGAA
38.	t3r14e	CTAATTTACCGTTTTTATTTTCATCTTGCGGG
39.	t3r14f	CAAGCAAGCGAGCATGTAGAAACCAGAGAATA
40.	t3r16e	ACGCTCAACGACAAAAGGTAAAGTATCCCATC
41.	t3r16f	TAAAGTACCAGTAGGGCTTAATTGCTAAATTT
42.	t3r18e	TATGTAAAGAAATACCGACCGTGTTAAAGCCA
43.	t3r18f	AATGGTTTTGCTGATGCAAATCCATTTTCCCT
44.	t3r20e	TTGAATTATTGAAAACATAGCGATTATAACTA
45.	t3r20f	TAGAATCCCCTTTTTTAATGGAAACGGATTCG
46.	t3r22e	ACAGAAATCTTTGAATACCAAGTTAATTTCAT
47.	t3r22f	CCTGATTGAAAGAAATTGCGTAGAAGAAGGAG
48.	t3r24e	CGACAACTTCATCATATTCCTGATCACGTAAA
49.	t3r24f	CGGAATTACGTATTAAATCCTTTGGTTGGCAA

50.	t3r26e	GCCACGCTTTGAAAGGAATTGAGGAAACAATT
51.	t3r26f	ATCAACAGGAGAGCCAGCAGCAAAATATTTTT
52.	t3r28e	GTCACACGATTAGTCTTTAATGCGGCAACAGT
53.	t3r28f	GAATGGCTACCAGTAATAAAAGGGCAAACTAT
54.	t3r2e	GGAAAGCGGTAACAGTGCCCGTATCGGGGTTT
55.	t3r2f	TGCCTTGACAGTCTCTGAATTTACCCCTCAGA
56.	t3r30e	GTAAAAGACTGGTAATATCCAGAAATTCACCA
57.	t3r30f	CGGCCTTGGTCTGTCCATCACGCATTGACGAG
58.	t3r32h	CACGTATAACGTGCTTTCCTCGTTGCCACCGA
59.	t3r4e	GTTTGCCACCTCAGAGCCGCCACCGCCAGAAT
60.	t3r4f	GCCACCACTCTTTTCATAATCAAATAGCAAGG
61.	t3r6e	TTATTCATGTCACCAATGAAACCATTATTAGC
62.	t3r6f	CCGGAAACTAAAGGTGAATTATCATAAAAGAA
63.	t3r8e	ATACCCAAACACCACGGAATAAGTGACGGAAA
64.	t3r8f	ACGCAAAGAAGAACTGGCATGATTTGAGTTAA
65.	t5r0g	CCTCAAGAGAAGGATTAGGATTAGAAACAGTT
66.	t5r10e	CTTTACAGTATCTTACCGAAGCCCAGTTACCA
67.	t5r10f	GCAATAGCAGAGAATAACATAAAAACAGCCAT
68.	t5r12e	GAGGCGTTTCCCAATCCAAATAAGATAGCAGC
69.	t5r12f	ATTATTTATTAGCGAACCTCCCGACGTAGGAA
70.	t5r14e	TAAGTCCTGCGCCCAATAGCAAGCAAGAACGC
71.	t5r14f	TCATTACCGAACAAGAAAAATAATAATTCTGT
72.	t5r16e	GCGTTATACGACAATAAACAACATACAATAGA
73.	t5r16f	CCAGACGACAAATTCTTACCAGTAGATAAATA
74.	t5r18e	TAACCTCCAATAAGAATAAACACCTATCATAT
75.	t5r18f	AGGCGTTAGGCTTAGGTTGGGTTAAGCTTAGA
76.	t5r20e	AAAACAAACTGAGAAGAGTCAATATACCTTTT
77.	t5r20f	TTAAGACGATTAATTACATTTAACACAAAATC

78.	t5r22e	AACCTACCGCGAATTATTCATTTCACATCAAG
79.	t5r22f	GCGCAGAGATATCAAAATTATTTGTATCAGAT
80.	t5r24e	GGATTTAGTTCATCAATATAATCCAGGGTTAG
81.	t5r24f	GATGGCAAAAGTATTAGACTTTACAAGGTTAT
82.	t5r26e	AGGCGGTCTCTTTAGGAGCACTAAACATTTGA
83.	t5r26f	CTAAAATAAGTATTAACACCGCCTCGAACTGA
84.	t5r28e	GAAATGGAAAACATCGCCATTAAACAGAGGTG
85.	t5r28f	TAGCCCTATTATTTACATTGGCAGCAATATTA
86.	t5r2e	ACAAACAACTGCCTATTTCGGAACCTGAGACT
87.	t5r2f	AATGCCCCATAAATCCTCATTAAAAGAACCAC
88.	t5r30e	AGAAGTGTCATTGCAACAGGAAAAAATCGTCT
89.	t5r30f	CCGCCAGCTTTTATAATCAGTGAGAGAATCAG
90.	t5r32h	AGCGGGAGCTAAACAGGAGGCCGAGAATCCTG
91.	t5r4e	TCGGCATTCCGCCGCCAGCATTGATGATATTC
92.	t5r4f	CACCAGAGTTCGGTCATAGCCCCCTCGATAGC
93.	t5r6e	ATTGAGGGAATCAGTAGCGACAGACGTTTTCA
94.	t5r6f	AGCACCGTAGGGAAGGTAAATATTTTATTTTG
95.	t5r8e	GAAGGAAAAATAGAAAATTCATATTTCAACCG
96.	t5r8f	TCACAATCCCGAGGAAACGCAATAATGAAATA
97.	t7r0f	TTTTTGAAAGTATTAAGAGGCTATTATT
98.	t7r10f	AAAAGTAATTTTAACGTCAAAAATGAAAAAACGATT
99.	t7r12f	TTTTGTTTTTTTGCTTATCCGGTATTCTAAATCAGA
100.	t7r14f	TATAGAAGTTTTACGCGCCTGTTTATCAGTTCAGCT
101.	t7r16f	AATGCAGATTTTGAAAAAGCCTGTTTAGGGAATCAT
102.	t7r18f	AATTACTATTTTCATAGGTCTGAGAGACGTGAATTT
103.	t7r20f	ATCAAAATTTTTGAAGATGATGAAACAAAATTACCT
104.	t7r22f	GAGCAAAATTTTACTTCTGAATAATGGATGATTGTT
105.	t7r24f	TGGATTATTTTTGCCGTCAATAGATAATCAACTAAT

106.	t7r26f	AGATTAGATTTTCCAGCAGAAGATAAAAAATACCGA
107.	t7r28f	ACGAACCATTTTCTACATTTTGACGCTCACGCTCAT
108.	t7r2f	CTGAAACATTTTGTCAGACGATTGGCCTCAGGAGGT
109.	t7r30j	GGAAATACTTTTCAGGAACGGTACGCCATTAAAGGGATTTTAGA
110.	t7r4f	TGAGGCAGTTTTGCGTCAGACTGTAGCGATCAAGTT
111.	t7r6f	TGCCTTTATTTTAGACAAAAGGGCGACAGGTTTACC
112.	t7r8f	AGCGCCAATTTTGCAGATAGCCGAACAATTTTTAAG
113.	t-1r0g	TATCACCGTACTCAGGAGGTTTAGATAGTTAG
114.	t-1r10e	GGACGTTGAGAACTGGCTCATTATGCGCTAAT
115.	t-1r10f	CGATTTTAGGAAGAAAAATCTACGGATAAAAA
116.	t-1r12e	TTTGCCAGGCGAGAGGCTTTTGCAATCCTGAA
117.	t-1r12f	CCAAAATAAGGGGGTAATAGTAAAAAAAGATT
118.	t-1r14e	TTTTAATTGCCCGAAAGACTTCAACAAGAACG
119.	t-1r14f	AAGAGGAACGAGCTTCAAAGCGAAAGTTTCAT
120.	t-1r16e	CGAGTAGAACAGTTGATTCCCAATATTTAGGC
121.	t-1r16f	TCCATATATTTAGTTTGACCATTAAGCATAAA
122.	t-1r18e	CTGTAATAGGTTGTACCAAAAACACAAATATA
123.	t-1r18f	GCTAAATCCTTTTGCGGGAGAAGCCCGGAGAG
124.	t-1r20e	TCAGGTCATTTTTGAGAGATCTACCCTTGCTT
125.	t-1r20f	GGTAGCTATTGCCTGAGAGTCTGGTTAAATCA
126.	t-1r22e	AAATAATTTTTAACCAATAGGAACAACAGTAC
127.	t-1r22f	GCTCATTTCGCGTCTGGCCTTCCTGGCCTCAG
128.	t-1r24e	GCTTCTGGCACTCCAGCCAGCTTTACATTATC
129.	t-1r24f	GAAGATCGTGCCGGAAACCAGGCAGTGCCAAG
130.	t-1r26e	CCCGGGTACCTGCAGGTCGACTCTCAAATATC
131.	t-1r26f	CTTGCATGCCGAGCTCGAATTCGTCCTGTCGT
132.	t-1r28e	GGGAGAGGCATTAATGAATCGGCCACCTGAAA
133.	t-1r28f	GCCAGCTGCGGTTTGCGTATTGGGAATCAAAA

134.	t-1r2e	ACGTTAGTTCTAAAGTTTTGTCGTGATACAGG
135.	t-1r2f	CGTAACGAAAATGAATTTTCTGTAGTGAATTT
136.	t-1r30e	AGTTTGGACGAGATAGGGTTGAGTGTAATAAC
137.	t-1r30f	GAATAGCCACAAGAGTCCACTATTAAGCCGGC
138.	t-1r32h	GAACGTGGCGAGAAAGGAAGGGAATGCGCCGC
139.	t-1r4e	CAATGACAGCTTGATACCGATAGTCTCCCTCA
140.	t-1r4f	CTTAAACAACAACCATCGCCCACGCGGGTAAA
141.	t-1r6e	AAACGAAATGCCACTACGAAGGCAGCCAGCAA
142.	t-1r6f	ATACGTAAGAGGCAAAAGAATACACTGACCAA
143.	t-1r8e	CCAGGCGCGAGGACAGATGAACGGGTAGAAAA
144.	t-1r8f	CTTTGAAAATAGGCTGGCTGACCTACCTTATG
145.	t-3r0g	CCCTCAGAACCGCCACCCTCAGAAACAACGCC
146.	t-3r10e	ACGAACTATTAATCATTGTGAATTTCATCAAG
147.	t-3r10f	TTTCAACTACGGAACAACATTATTAACACTAT
148.	t-3r12e	ACTGGATATCGTTTACCAGACGACTTAATAAA
149.	t-3r12f	CATAACCCGCGTCCAATACTGCGGTATTATAG
150.	t-3r14e	GAAGCAAAAAAGCGGATTGCATCAATGTTTAG
151.	t-3r14f	TCAGAAGCCTCCAACAGGTCAGGATTTAAATA
152.	t-3r16e	TCGCAAATAAGTACGGTGTCTGGACCAGACCG
153.	t-3r16f	TGCAACTAGGTCAATAACCTGTTTAGAATTAG
154.	t-3r18e	CAACGCAAAGCAATAAAGCCTCAGGATACATT
155.	t-3r18f	CAAAATTAGGATAAAAATTTTTAGGATATTCA
156.	t-3r20e	AGAGAATCAGCTGATAAATTAATGCTTTATTT
157.	t-3r20f	ACCGTTCTGATGAACGGTAATCGTAATATTTT
158.	t-3r22e	CTTTCATCTCGCATTAAATTTTTGAGCAAACA
159.	t-3r22f	GTTAAAATAACATTAAATGTGAGCATCTGCCA
160.	t-3r24e	TTCGCCATGGACGACGACAGTATCGTAGCCAG
161.	t-3r24f	GTTTGAGGTCAGGCTGCGCAACTGTTCCCAGT

162.	t-3r26e	TCATAGCTTGTAAAACGACGGCCAAAGCGCCA
163.	t-3r26f	CACGACGTGTTTCCTGTGTGAAATTTGCGCTC
164.	t-3r28e	TGGTTTTTCTTTCCAGTCGGGAAAAATCATGG
165.	t-3r28f	ACTGCCCGCTTTTCACCAGTGAGATGGTGGTT
166.	t-3r2e	TGCTAAACTCCACAGACAGCCCTCTACCGCCA
167.	t-3r2f	TGTAGCATAACTTTCAACAGTTTCTAATTGTA
168.	t-3r30e	TGGACTCCGGCAAAATCCCTTATACGCCAGGG
169.	t-3r30f	CCGAAATCAACGTCAAAGGGCGAAAAGGGAGC
170.	t-3r32h	CCCCGATTTAGAGCTTGACGGGGAAAAGAACG
171.	t-3r4e	ATATATTCTCAGCTTGCTTTCGAGTGGGATTT
172.	t-3r4f	TCGGTTTAGGTCGCTGAGGCTTGCAAAGACTT
173.	t-3r6e	CTCATCTTGGAAGTTTCCATTAAACATAACCG
174.	t-3r6f	TTTCATGATGACCCCCAGCGATTAAGGCGCAG
175.	t-3r8e	AGTAATCTTCATAAGGGAACCGAACTAAAACA
176.	t-3r8f	ACGGTCAATGACAAGAACCGGATATGGTTTAA
177.	t-5r0g	CTCAGAGCCACCACCCTCATTTTCCGTAACAC
178.	t-5r10e	AAAGATTCTAAATTGGGCTTGAGATTCATTAC
179.	t-5r10f	ACGAGTAGATCAGTTGAGATTTAGCGCCAAAA
180.	t-5r12e	TAAATATTGAGGCATAGTAAGAGCACAGGTAG
181.	t-5r12f	GGAATTACCATTGAATCCCCCTCACCATAAAT
182.	t-5r14e	TACCTTTAAGGTCTTTACCCTGACAATCGTCA
183.	t-5r14f	CAAAAATCATTGCTCCTTTTGATAATTGCTGA
184.	t-5r16e	TTTCATTTCTGTAGCTCAACATGTTTAGAGAG

185.	t-5r16f	ATATAATGGGGGCGCGAGCTGAAATTAACATC
186.	t-5r18e	TATATTTTCATACAGGCAAGGCAAAGCTATAT
187.	t-5r18f	CAATAAATAAATGCAATGCCTGAGAAGGCCGG
188.	t-5r20e	CATGTCAAAAATCACCATCAATATAACCCTCA
189.	t-5r20f	AGACAGTCTCATATGTACCCCGGTTTGTATAA
190.	t-5r22e	ACCCGTCGTTAAATTGTAAACGTTAAAACTAG
191.	t-5r22f	GCAAATATGATTCTCCGTGGGAACCGTTGGTG
192.	t-5r24e	GGCGATCGCGCATCGTAACCGTGCGAGTAACA
193.	t-5r24f	TAGATGGGGTGCGGGCCTCTTCGCGCAAGGCG
194.	t-5r26e	GCTCACAAGGGTAACGCCAGGGTTTTGGGAAG
195.	t-5r26f	ATTAAGTTTTCCACACAACATACGCCTAATGA
196.	t-5r28e	AGCTGATTACTCACATTAATTGCGTGTTATCC
197.	t-5r28f	GTGAGCTAGCCCTTCACCGCCTGGGGTTTGCC
198.	t-5r2e	GAGAATAGGTCACCAGTACAAACTCCGCCACC
199.	t-5r2f	TGAGTTTCAAAGGAACAACTAAAGATCTCCAA
200.	t-5r30e	TATCAGGGCGAAAATCCTGTTTGACGGGCAAC
201.	t-5r30f	CCAGCAGGCGATGGCCCACTACGTGAGGTGCC
202.	t-5r32h	GTAAAGCACTAAATCGGAACCCTAAAACCGTC
203.	t-5r4e	AAAGGCCGCTCCAAAAGGAGCCTTAGCGGAGT
204.	t-5r4f	AAAAAAGGCTTTTGCGGGATCGTCGGGTAGCA

205.	t-5r6e	GCGAAACAAGAGGCTTTGAGGACTAGGGAGTT
206.	t-5r6f	ACGGCTACAAGTACAACGGAGATTCGCGACCT
207.	t-5r8e	CCAAATCATTACTTAGCCGGAACGTACCAAGC
208.	t-5r8f	GCTCCATGACGTAACAAAGCTGCTACACCAGA
209.	t-7r10e	CATTCAACTTTTCTTGCCCTGACGAGAACATTCAGT
210.	t-7r12e	AAACAGTTTTTTTAATGCAGATACATAAGAATACCA
211.	t-7r14e	TTTTTGCGTTTTCAGAAAACGAGAATGAAATGCTTT
212.	t-7r16e	TCAATTCTTTTTGATGGCTTAGAGCTTAAGAGGTCA
213.	t-7r18e	AGGTAAAGTTTTACTAATAGTAGTAGCAAGGTGGCA
214.	t-7r20e	AGAAAAGCTTTTATTCAAAAGGGTGAGATAATGTGT
215.	t-7r22e	GATTGACCTTTTCCCAAAAACAGGAAGATGATAATC
216.	t-7r24e	CAGCTGGCTTTTGTAATGGGATAGGTCAAAACGGCG
217.	t-7r26e	GCATAAAGTTTTGAAAGGGGGATGTGCTTATTACGC
218.	t-7r28e	GAGTTGCATTTTTGTAAAGCCTGGGGTGAGCCGGAA
219.	t-7r2i	AATAATAATTTTATAGGAACCCATGTACAGGGATAGCAAGCCCA
220.	t-7r30e	ACCCAAATTTTTGCAAGCGGTCCACGCTCCCTGAGA
221.	t-7r32e	TTTTCAAGTTTTTTGGGGTCGAACCATC
222.	t-7r4e	CAGCGAAATTTTTTTTTTCACGTTGAAAGAATTGCG
223.	t-7r6e	CGCCTGATTTTTGACAGCATCGGAACGAACCCTCAG
224.	t-7r8e	GAATAAGGTTTTAAATTGTGTCGAAATCTGTATCAT

225.	tr-rem1	GCGCTTAA

Table S2. Modified staple sequences to be used as capture strands for AuNPs attachment

S.No.	Sequence code	Sequence (5'-3')
226	Binding-1-1	A24+CCCTCAGAACCGCCACCCTCAGAAACAACGCC
227	Binding-1-2	TGCTAAACTCCACAGACAGCCCTCTACCGCC+A24
228	Binding-2-1	CTCAGAGCCACCACCCTCATTTTCCGTAACAC+A24
229	Binding-2-2	A24+TGAGTTTCAAAGGAACAACTAAAGATCTCCAA
230	Binding-3-1	A24+CGCCTGATGACAGCATCGGAACGAACCCTCAG
231	Binding-3-2	GAATAAGGAAATTGTGTCGAAATCTGTATCAT+A24
232	Binding-4-1	A24+AAACAGTTTAATGCAGATACATAAGAATACCA
233	Binding-4-2	TTTTTGCGCAGAAAACGAGAATGAAATGCTTT+A24
234	Binding-5-1	A24+AGGTAAAGACTAATAGTAGTAGCAAGGTGGCA
235	Binding-5-2	AGAAAAGCATTCAAAAGGGTGAGATAATGTGT+A24
236	Binding-6-1	A24+CAGCTGGCGTAATGGGATAGGTCAAAACGGCG
237	Binding-6-2	GCATAAAGGAAAGGGGGATGTGCTTATTACGC+A24
238	Binding-7-1	CCAGCAGGCGATGGCCCACTACGTGAGGTGCC+A24
239	Binding-7-2	A24+GTAAAGCACTAAATCGGAACCCTAAAACCGTC
240	Binding-8-1	A24+TGGACTCCGGCAAAATCCCTTATACGCCAGGG
241	Binding-8-2	CCCCGATTTAGAGCTTGACGGGGAAAAGAACG+A24
242	AuNP-sec1	/ 5ThioMC3-D / TTTTT TTTTTTTTTTTTTTTTTTTTTTTT
243	AuNP-sec2	TTTTTTTTTTTTTTTTTTTTTTTT TTTTT/3ThioMC3-D/

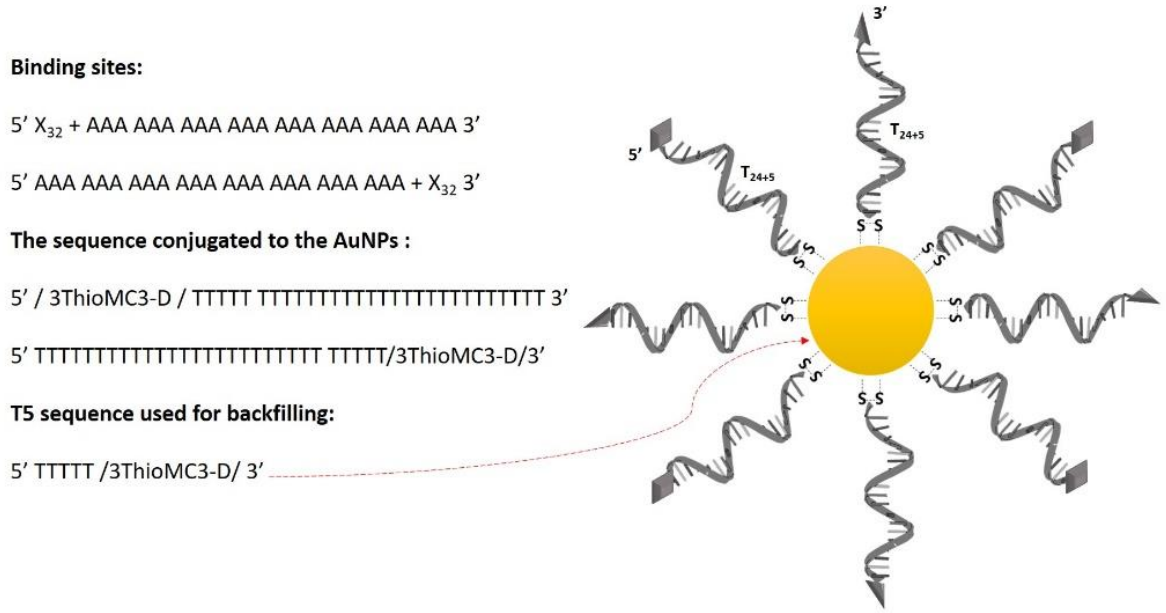

Figure S2 Design of extended binding strands on the surface and corresponding complementary strands on the functionalized gold nanoparticles. The strands on neighboring sites do not contend each other, since they are designed in opposite directions. The specific length and sequences of the 8 chosen binding sites (2 staple strands per site) are listed in Table S2.

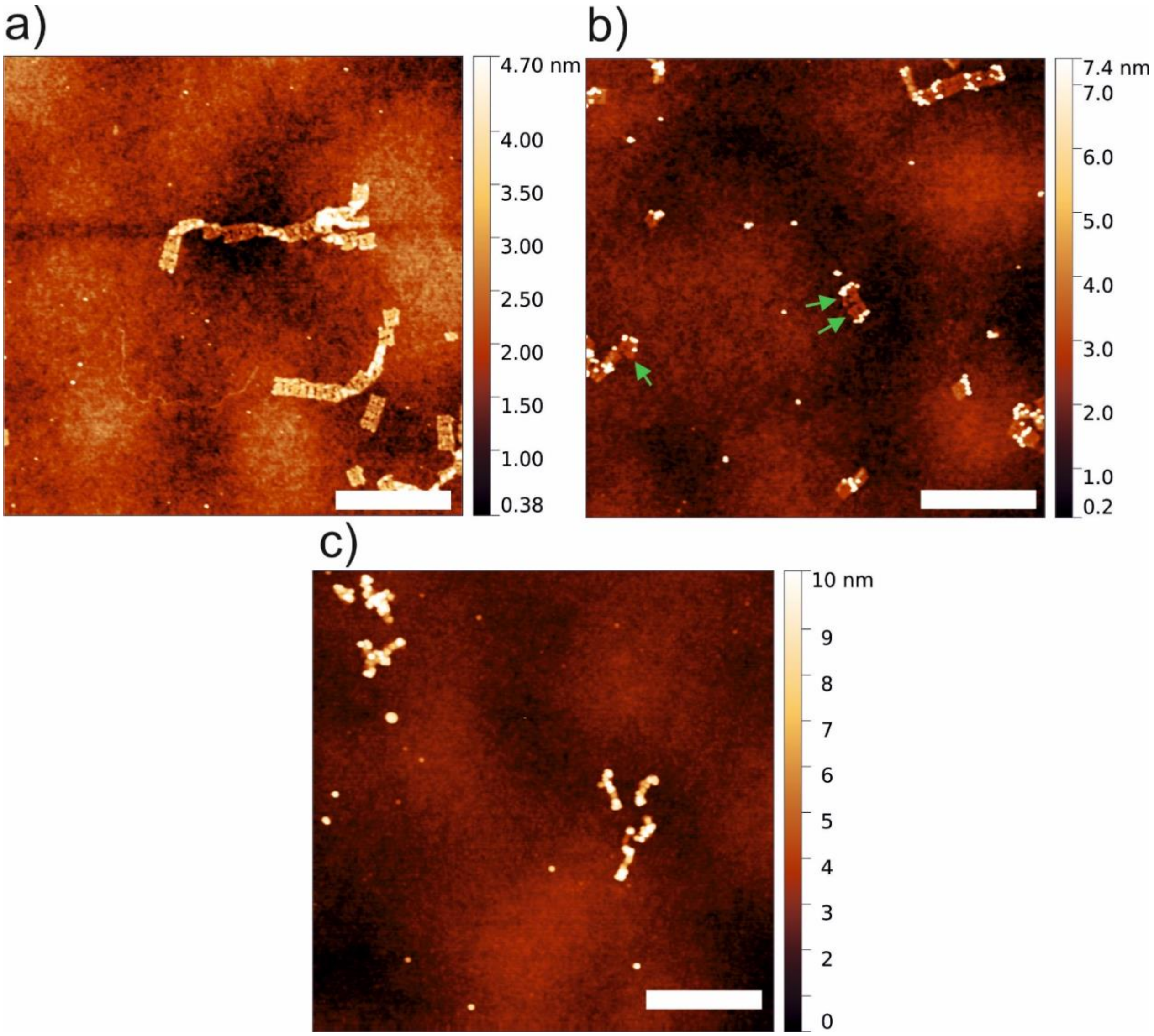

Figure S3. AFM height images of a) bare DNA origami and b) − c) AuNP decorated DNA origami nanosheets laid out on a SiO_2 surface. Defects, deformations, pi-pi stacking interactions between adjacent nanosheeets were observed in some samples before and after the AuNP attachment process, as shown in figures a) and b), which may hinder the high yield of AuNP attachment. Defects are highlighted by green arrows on figure b). In figure c), some nanosheets are observed to be folded. The unfunctionalized side lies on the surface, which contains the capture strands, hindering Au NP attachment during the hybridization. The scale bars are 500 nm.

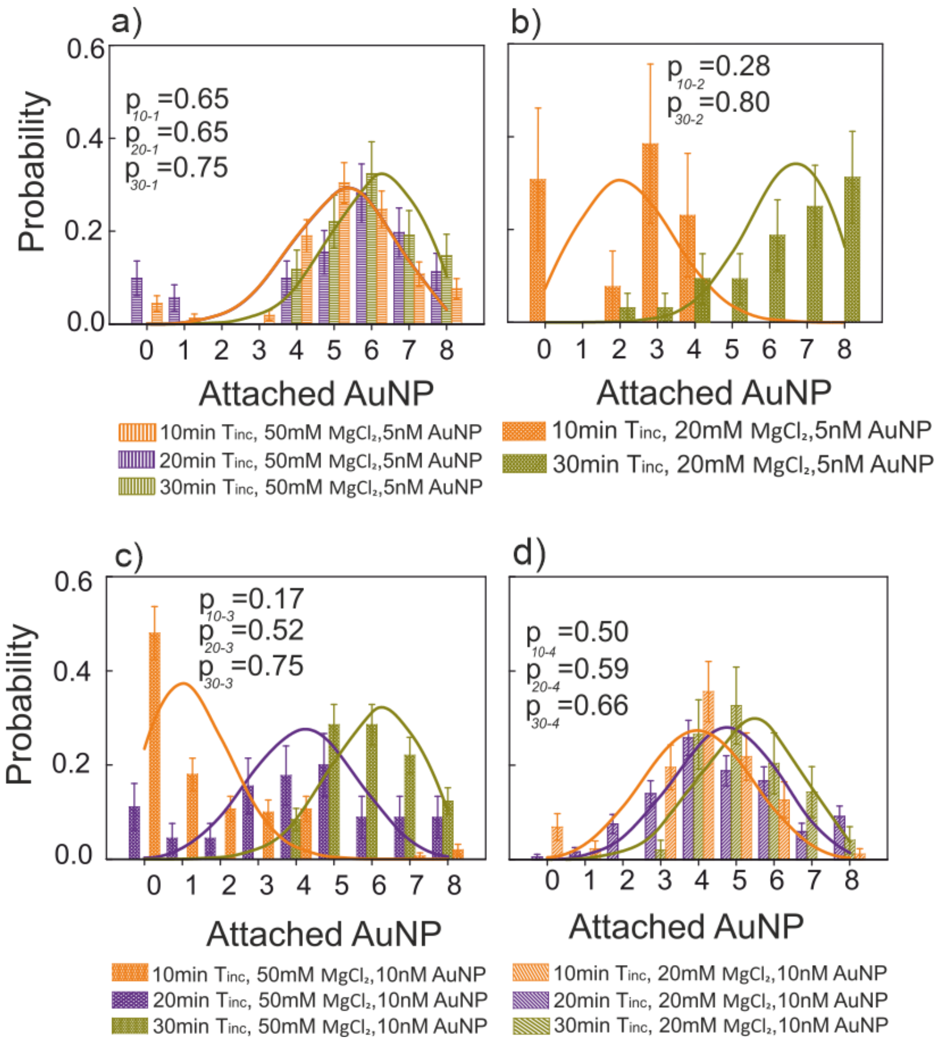

Figure S4. Histograms obtained from AFM images and expected binomial distributions (solid lines) are used to determine the attachment probabilities of AuNPs for several experimental conditions, as shown below each graph. On figure a), the histograms peaks were found at six successful attachments when 50 mM $MgCl_2$ and 2.5:1of functionalized AuNP:DNA molar ratio for T_{inc} = 20 and 30 min. At lower $MgCl_2$ concentration (20 mM), the number of successful attached AuNP increased to eight for T_{inc} = 30min, as observed in figure b). On figure c), the histogram shows two peaks at 5 (20 min) and 6 (30 min) successful attachments at high $MgCl_2$ and AuNP:DNA concentrations. If the $MgCl_2$ concentration varies to 20 mM, keeping the functionalized AuNP:DNA molar ratio at 5:1, the number of successful attached AuNP decreased to 4 and 5, as shown in figure d). The histograms also show that there is a low yield of attached AuNPs, for T_{inc} = 10 min. An increase of functionalized AuNP:DNA molar ratio from 2.5:1 to 5:1 does not change the number of successful attachments. Besides, it results in a very large density of unbound background AuNPs and some of these AuNPs form clusters and appear larger than the physical size of individual AuNPs, as observed from AFM images. AFM images showed that the unattached sites tend to fold towards the nanoparticle attached sites of the origami nanosheets while each AuNP:DNA origami conjugate formed a C-shape nanowire for T_{inc} = 20 min, 20 mM $MgCl_2$ concentration and 5:1 molar ratio of AuNP:DNA, as seen in figure S2 c). In

this work, the origami template was designed with only two capture strands per binding site and a site-spacing of only 16nm. The low amount of capture stands and small distance between consecutive sites may increase the impact of steric hindrance and electrostatic repulsion. Overall, these effects elucidate the low values of the distribution probabilities. The individual AuNPs bound to multiple binding sites tend to aggregate at high concentrations and, as a consequence, the origami is flexible on SiO_2/Si. Thus, unattached sites of the origami have a tendency to fold onto a nanoparticle attached to another site and it is difficult to achieve a C-shaped formation attachment of AuNPs. Therefore, to study the electrical properties of C-shape gold nanowires templated by DNA origami we fabricated this structure using the experimental conditions with the highest probability, $p = 0.80$, of AuNPs attachment.

Table S3. Summary of the AuNP attachment probability obtained from the statistical analysis for different incubation times (T_{inc}), $MgCl_2$ concentrations (mM) and AuNP:DNA origami molar ratio.

**Label**	**T_{inc} (min)**	**$MgCl_2$ conc. (nM)**	**AuNP:DNA Orig.**	**Probability**
P_{10-1}	10	50	2.5:1	0.65
P_{20-1}	20	50	2.5:1	0.65
P_{30-1}	30	50	2.5:1	0.75
P_{10-2}	10	20	2.5:1	0.28
P_{30-2}	30	20	2.5:1	0.80
P_{10-3}	10	50	5:1	0.17
P_{20-3}	20	50	5:1	0.52
P_{30-3}	30	50	5:1	0.75
P_{10-4}	10	20	5:1	0.50
P_{20-4}	20	20	5:1	0.59
P_{30-4}	30	20	5:1	0.66

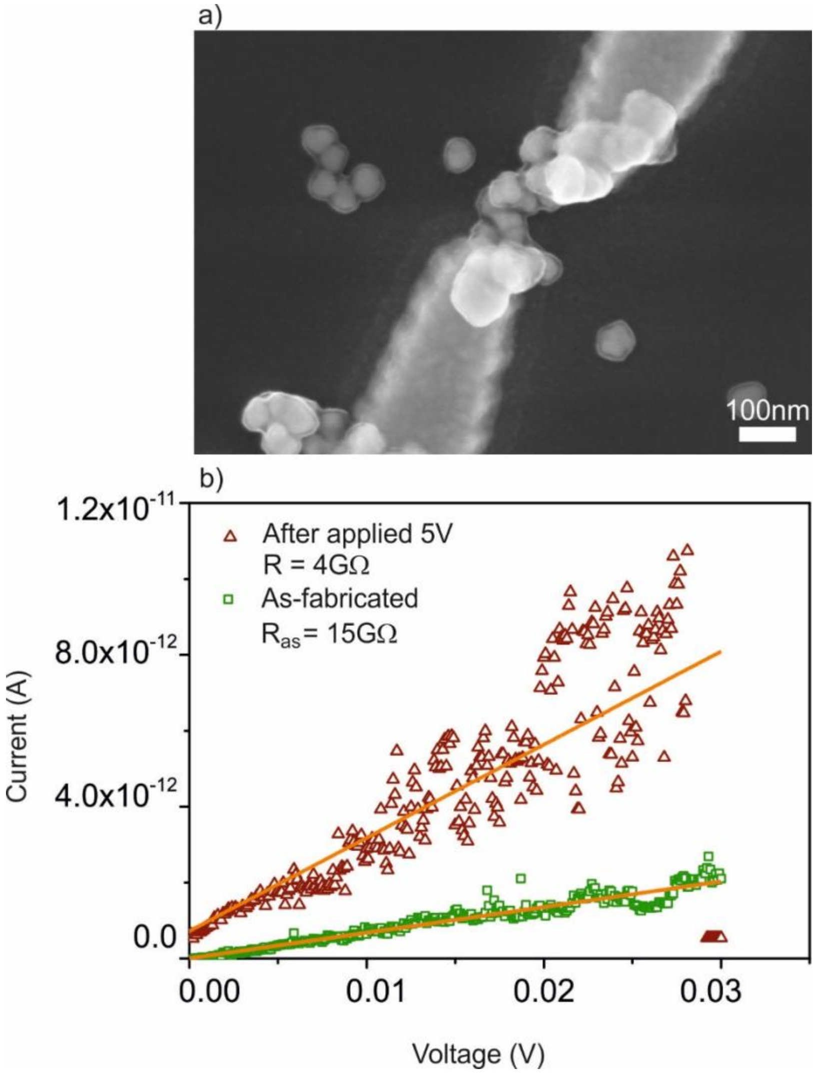

Figure S5. (a) SEM image of a C-shape wire between electrodes. (b) Current-Voltage curves in the 0-30 mV voltage range of the as-fabricated wire (green squares) shown in a) and after applying different bias voltages up to 5V on the same wire (red triangles). Resistance of the as-fabricated wire resulted to be 15GΩ at 30 mV, while the resistance was found to be 4 GΩ after increasing the applied bias voltage bias form 30mV, 50mV, 1V, 2V, 3V, 5V and then back to 30mV.

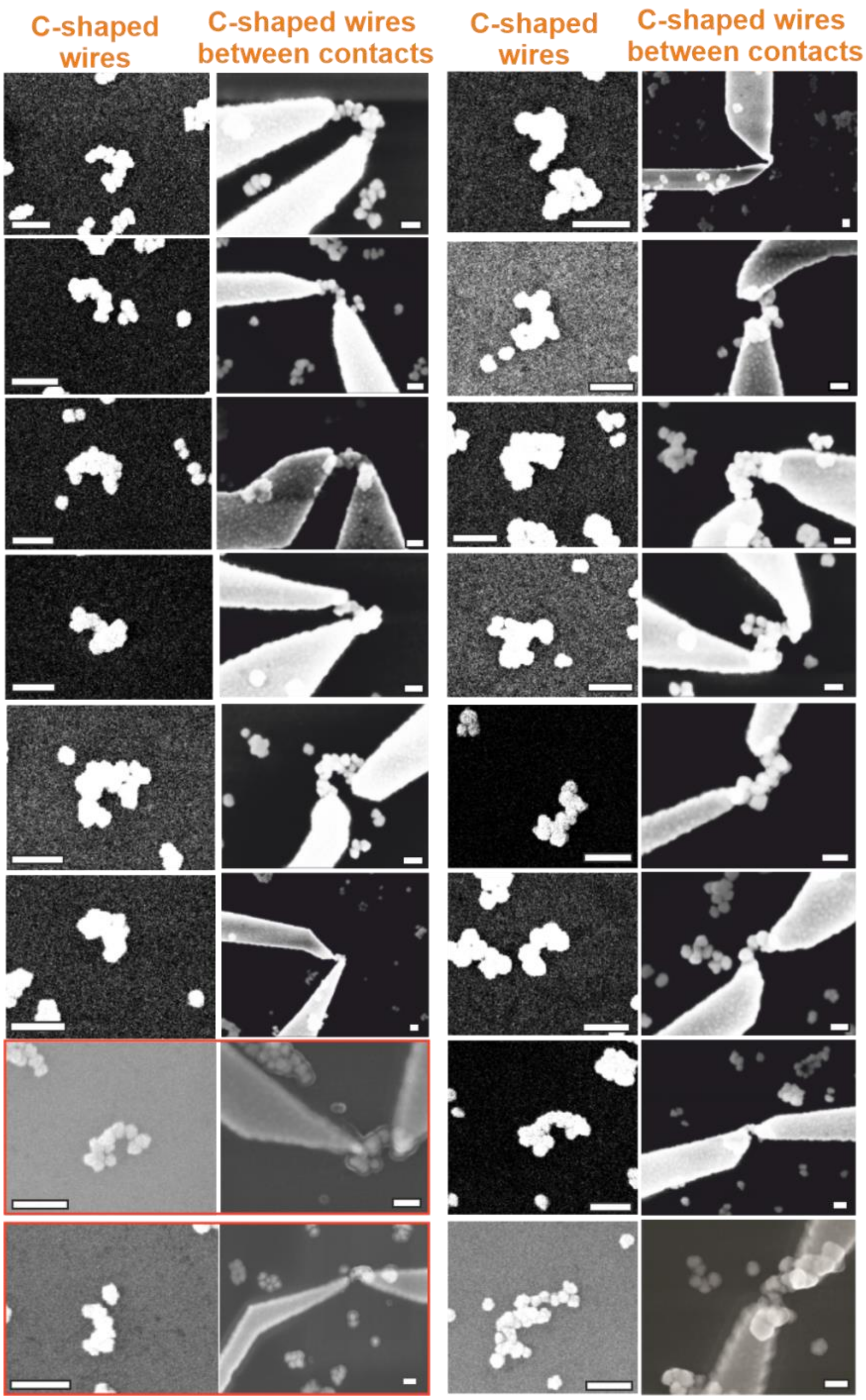

Figure S6. SEM images of the 16 C-shape nanowires before and after being contacted by Au electrodes. The images framed in red correspond to the nanowires used for the temperature-dependent *I-V* characterization in the voltage range between -30 and 30mV and shown in figure 2 f). SEM imaging revealed 1−3 nm gaps between the contact points leading to high resistance in wires. Scale bars are is 250nm for C-shaped wires and 100nm for NW with contacts in SEM images.

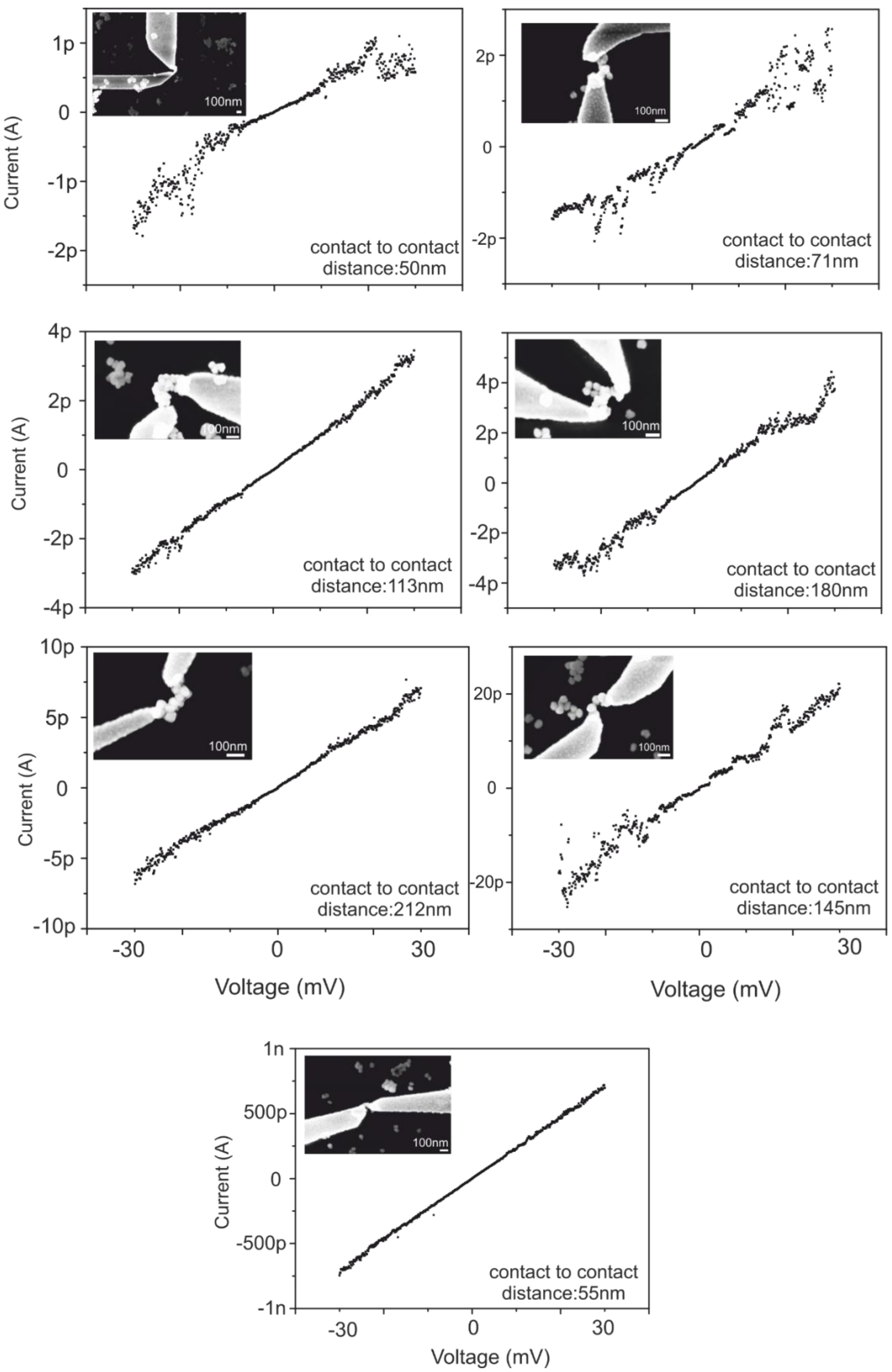

Current (A)
1p
0
-1p
-2p
100nm
contact to contact
distance:50nm
2p
0
-2p
100nm
contact to contact
distance:71nm
4p
2p
0
-2p
-4p
100nm
contact to contact
distance:113nm
4p
2p
0
-2p
-4p
100nm
contact to contact
distance:180nm
10p
5p
0
-5p
-10p
100nm
contact to contact
distance:212nm
20p
0
-20p
100nm
contact to contact
distance:145nm
-30
0
30
Voltage (mV)
1n
500p
0
-500p
-1n
100nm
contact to contact
distance:55nm

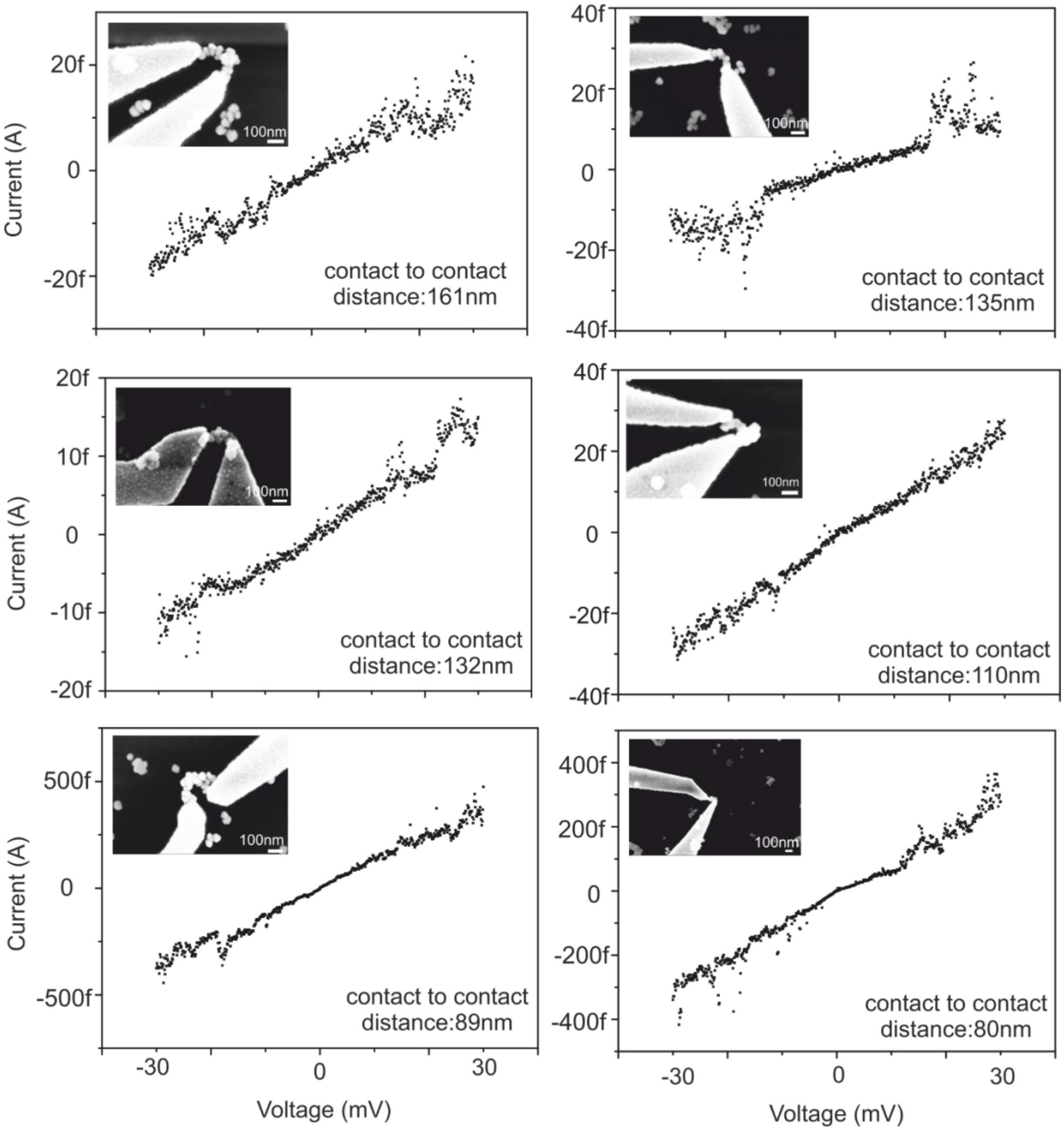

Figure S7. *I-V* characterization of 13, out of 16, contacted resistive wires, as shown in figure S6, at RT in high vacuum and darkness. Two *I-V* curves left out here are already shown in figure 3 d)−e). The third curve, which corresponds to the contacted wire shown on the bottom right of figure S5, is missing because its electrical characteristics showed a short circuit.

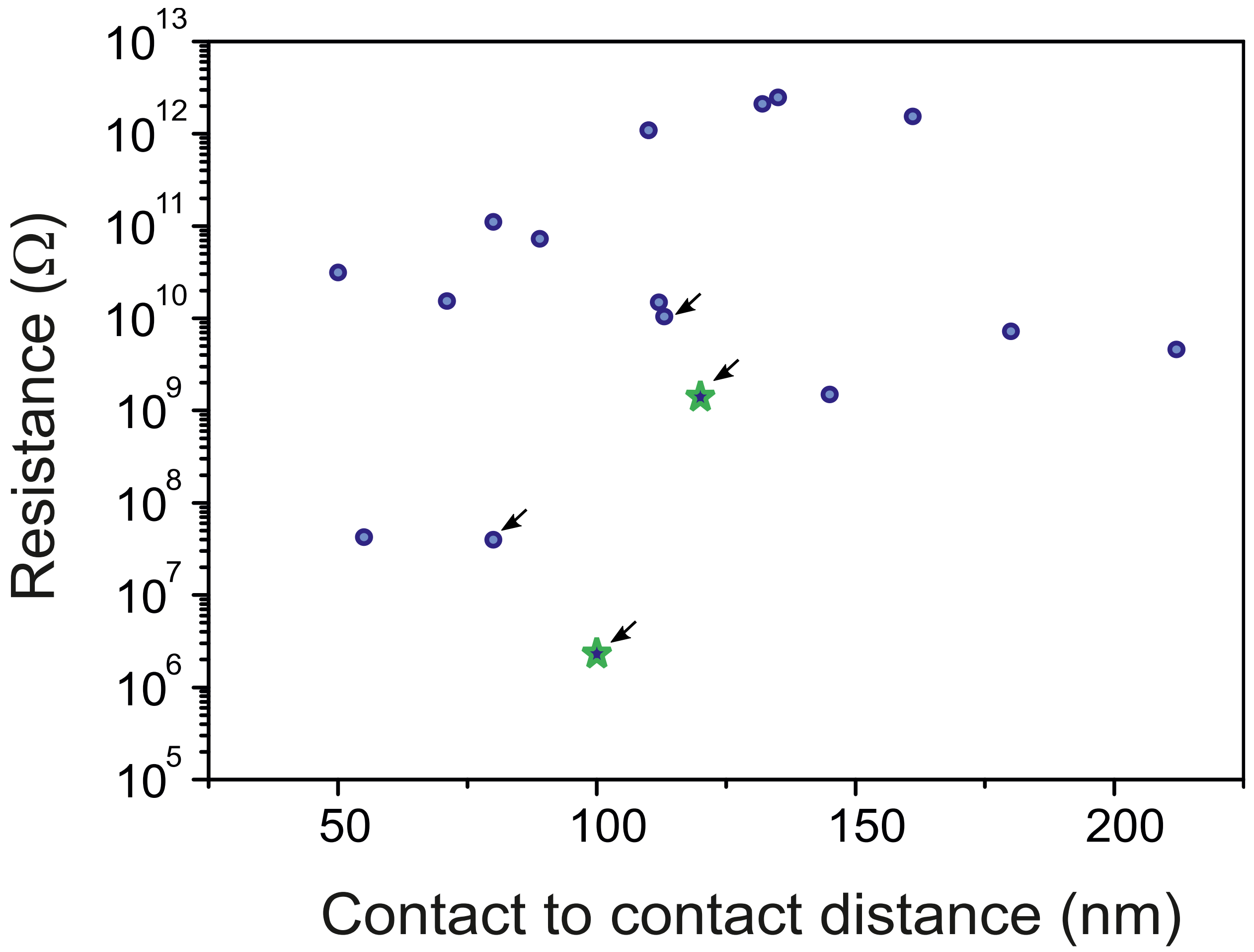

Figure S8. The resistance values shown here were obtained from linear fits in the -10 to 10 mV voltage range from the corresponding graphs in figures 3 d)−e) and S7. The resistance values as a function of contact to contact distance between the EBL-patterned gold electrodes for the characterized gold nanowires. Wires chosen for temperature-dependent measurements are shown by a star-shaped symbol. The arrows indicate wires which were not treated with oxygen plasma before *IV*-measurement, all others were. There is no systematic difference seen between these wires, therefore we conclude that the influence of carbon deposits on the wires, which happens during EBL, can be neglected.

OBSERVATIONS. The room temperature resistances of NW-1 and NW-2 are lower than those NWs fabricated on DNA origami nanotubes (the measured resistances were 116 MΩ and 2.8 GΩ at RT from a previous report (26)). The NWs fabricated using DNA origami nanotube templates are 3× longer than the NWs reported here but they have the same width (26). Four-probe electrical characterization for anisotropically grown gold NWs utilizing the same DNA origami template (nanosheet) but attaching gold nanorods instead of AuNPs has demonstrated resistance values between 0.4 kΩ to 10 GΩ (29). On the contrary, Tian *et al.* have measured highly resistive DNA origami nanopillar arrays (28). Other DNA origami templated gold NWs have reported lower resistance values than those measured at RT here (18,21,22,27). The production of continuous and smooth gold nanowires having a low resistance with ohmic behavior strongly depends on each of the stages of the synthesis.
